# Antifouling All‐Polymeric Microneedle Array for Long‐Term Wearable ECG Monitoring

**DOI:** 10.1002/advs.202512430

**Published:** 2025-11-14

**Authors:** Ju Hyeon Kim, Chuljin Hwang, Jee Hoon Lee, Hang Chan Jo, Dae Yu Kim

**Affiliations:** ^1^ Department of Mechanical Engineering Inha University Incheon 22212 Republic of Korea; ^2^ Department of Electrical and Computer Engineering Inha University Incheon 22212 Republic of Korea; ^3^ Inha Research Institute for Aerospace Medicine Inha University Incheon 22212 Republic of Korea; ^4^ Center for Sensor Systems Inha University Incheon 22212 Republic of Korea

**Keywords:** all‐polymeric microneedle, antifouling, electrocardiogram, microneedle, zwitterionic polymer

## Abstract

Cardiovascular disease demands reliable long‐term monitoring for early and accurate diagnosis. While conventional gel electrodes are commonly utilized for monitoring electrocardiogram (ECG) signals, they have limitations, such as dehydration‐induced signal degradation and motion artifacts, hindering their effectiveness for continuous long‐term use. To address these issues, this study develops a biocompatible and all‐polymeric microneedle electrode (MNE) optimized for stable ECG monitoring over extended periods. The MNE surface is sequentially coated with poly(3,4‐ethylenedioxythiophene):tosylate (PEDOT:Tos) to reduce the interfacial impedance (0.63 kΩ∙cm^2^ at 10 Hz), followed by zwitterionic sulfobetaine methacrylate (SBMA) to prevent nonspecific protein adsorption and cellular adhesion (84.2% and 98.6% reduction in E. coli and BSA, respectively). Moreover, the SBMA‐coated MNE demonstrates excellent mechanical strength, allowing it to penetrate human skin without deformation, as verified by optical coherence tomography and insertion‐force assessments. In practical applications using a wireless wearable ECG monitoring system, the SBMA‐coated MNE consistently captures high‐quality ECG signals over a 14‐day period, significantly outperforming gel electrodes under dynamic movement conditions.

## Graphical abstract

A biocompatible, all‐polymeric MNEs designed for long‐term ECG monitoring is developed. Coated with PEDOT:Tos and zwitterionic SBMA, the MNEs offer ultra‐low impedance and antifouling properties. After 14 days of continuous use, the MNEs accurately detected PQRST complexes, highlighting its potential for long‐term, real‐time ECG monitoring in everyday life.

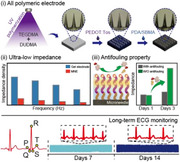

## 1 Introduction

Cardiovascular disease (CVD)—the leading global cause of death—accounted for ≈20.5 million deaths in 2021 and exhibits increasing prevalence across all age groups in various countries.^[^ ^1^, ^2^, ^3^, ^4^, ^5^, ^6^ ^]^ Early‐stage symptoms of CVD are often mild or intermittent, making accurate and timely diagnosis challenging, which increases the risk of delayed or missed intervention.^[^ ^7^, ^8^ ^]^ These clinical limitations underscore the necessity for real‐time cardiac monitoring capable of continuous, long‐term data acquisition beyond the short‐term assessments typically conducted in clinical settings.^[^ ^9^ ^]^

The electrocardiogram (ECG) serves as a crucial monitoring tool widely used in medical studies to provide direct insights into cardiac function and abnormalities, aiding the timely diagnosis and treatment of CVD.^[^ ^10^, ^11^, ^12^ ^]^ Conventionally, gel electrodes have been the gold standard for ECG monitoring in both clinical and research settings owing to their affordability and reliable signal quality.^[^ ^13^ ^]^ However, gel electrodes face challenges, such as signal degradation as the gel dehydrates, limiting continuous long‐term ECG monitoring.^[^ ^14^, ^15^ ^]^ Furthermore, they are prone to motion artifacts owing to weak mechanical coupling at the electrode–skin interface, resulting in relative displacement during movement.^[^ ^16^, ^17^ ^]^ Additionally, their prolonged use can result in skin irritation, inflammation, allergic reactions, and discomfort.^[^ ^18^, ^19^, ^20^ ^]^

To address these limitations, microneedle electrodes (MNEs) have emerged as a promising alternative because of their low electrode–skin interfacial impedance, reduced motion artifacts, and stable electrical performance without a conductive gel.^[^ ^21^, ^22^, ^23^, ^24^, ^25^ ^]^ In addition, their ability to penetrate the outermost layer of the skin allows direct access to the epidermis, forming a stable electrical interface without surface preparation.^[^ ^26^ ^]^ Solid substrates such as metals and silicon, characterized by a high Young's modulus (>100 GPa), provide mechanical robustness.^[^ ^27^, ^28^ ^]^ In contrast, polymeric substrates offer enhanced flexibility and conformability to the skin surface, which is advantageous for ensuring comfort and stability during long‐term biopotential monitoring.^[^ ^29^, ^30^ ^]^ For example, Gwak et al.^[^ ^31^ ^]^ reported the fabrication of metal MAEs for ECG detection using a low‐melting‐point Bi–In–Sn alloy, demonstrating sufficient mechanical robustness for skin penetration, stable electrode–skin interface impedance after polyurethane acrylate coating, and ECG recording performance comparable to that of conventional Ag/AgCl electrodes. In addition to rigid metal‐based designs, flexible approaches have been explored. Xing et al.^[^ ^32^ ^]^ developed a durable MNE with vertically aligned gold nanowires embedded in a polyimide substrate, resulting in a high signal‐to‐noise ratio (SNR) and superior ECG signal quality compared with gel electrodes. Similarly, Srivastava et al.^[^ ^33^ ^]^ introduced a low‐cost dry‐electrode system utilizing polymeric MNEs for biopotential recording, demonstrating signal quality comparable to that of gel electrodes, even after 48 h of continuous use.

However, despite these advancements, developing MNEs capable of continuous ECG monitoring with high sensitivity and long‐term reliability during daily activities remains challenging. The primary issue lies in a loss of sensitivity due to the formation of biofilms and biofouling resulting from the nonspecific adsorption of biomolecules onto the MNE surface over prolonged exposure to biological environments. These surface interactions lead to increased electrode–skin interface impedance and elevated noise levels, reducing the SNR and compromising the quality of the recorded ECG.^[^ ^34^ ^]^ Therefore, to ensure long‐term ECG monitoring, a robust surface coating strategy that minimizes the nonspecific biomolecular adsorption and biofilm formation is required.

**Figure 1:**
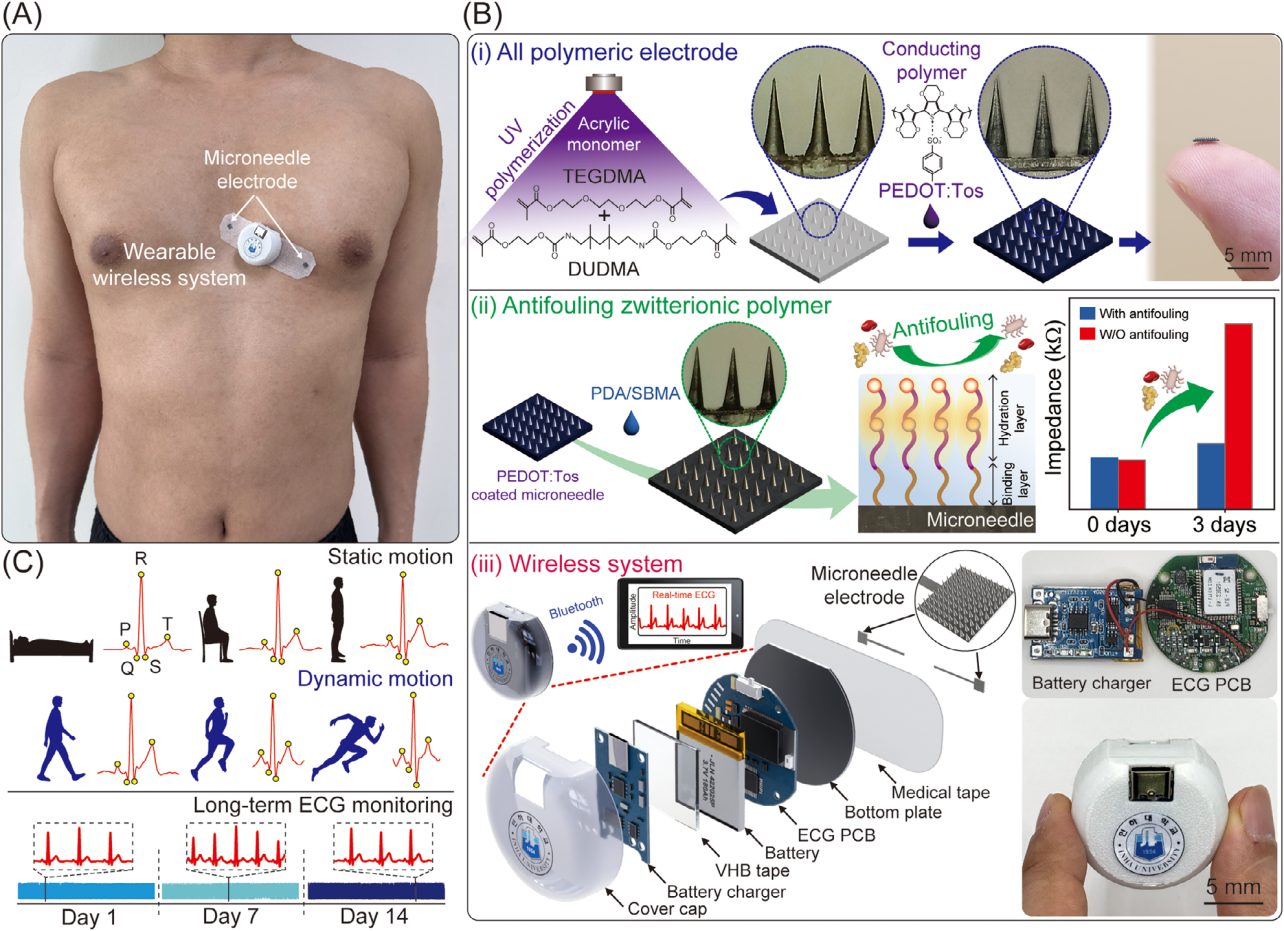
Overall design of the wireless ECG monitoring system. A) Photograph of a person wearing the ECG monitoring system. B) (i) Schematic of the process flow for fabricating all‐polymeric MNEs. (ii) Antifouling surface modification through the zwitterionic polymer. (iii) Cross‐section of the wearable wireless system with the MNE. C) Long‐term ECG monitoring under static and dynamic motion.

In this study, we developed skin‐integrated, biocompatible, and mechanically robust all‐polymeric MNEs for continuous ECG monitoring over a 14‐day period under diverse motion scenarios (**Figure** **1**A). The MNEs were fabricated using a composite monomer of triethylene glycol dimethacrylate (TEGDMA) and diurethane dimethacrylate (DUDMA) and subsequently functionalized with poly(3,4‐ethylenedioxythiophene):tosylate (PEDOT:Tos) to achieve an ultra‐low electrode–skin interfacial impedance (Figure 1B). Zwitterionic sulfobetaine methacrylate (SBMA) was coated onto the MNE surface via a one‐step self‐polymerization process, using polydopamine (PDA) as an adhesive linker. The antifouling nature of SBMA facilitated the formation of a stable hydration shell, effectively suppressing nonspecific biomolecular adsorption. Electrochemical impedance spectroscopy (EIS) validated the antifouling capabilities of the zwitterionic SBMA coating, which demonstrated sustained impedance density even after prolonged use. Finally, the MNEs were integrated into a wearable wireless system consisting of a tablet‐based ECG monitoring platform, custom‐designed integrated circuits (ICs), and an Android application for real‐time ECG visualization. Even after continuous use for 14 days, our system demonstrated the ability to accurately detect PQRST waveforms with high fidelity (Figure 1C).

## 2 Results

### 2.1 Antifouling Test with SBMA‐Coated MNEs

**Figure 2:**
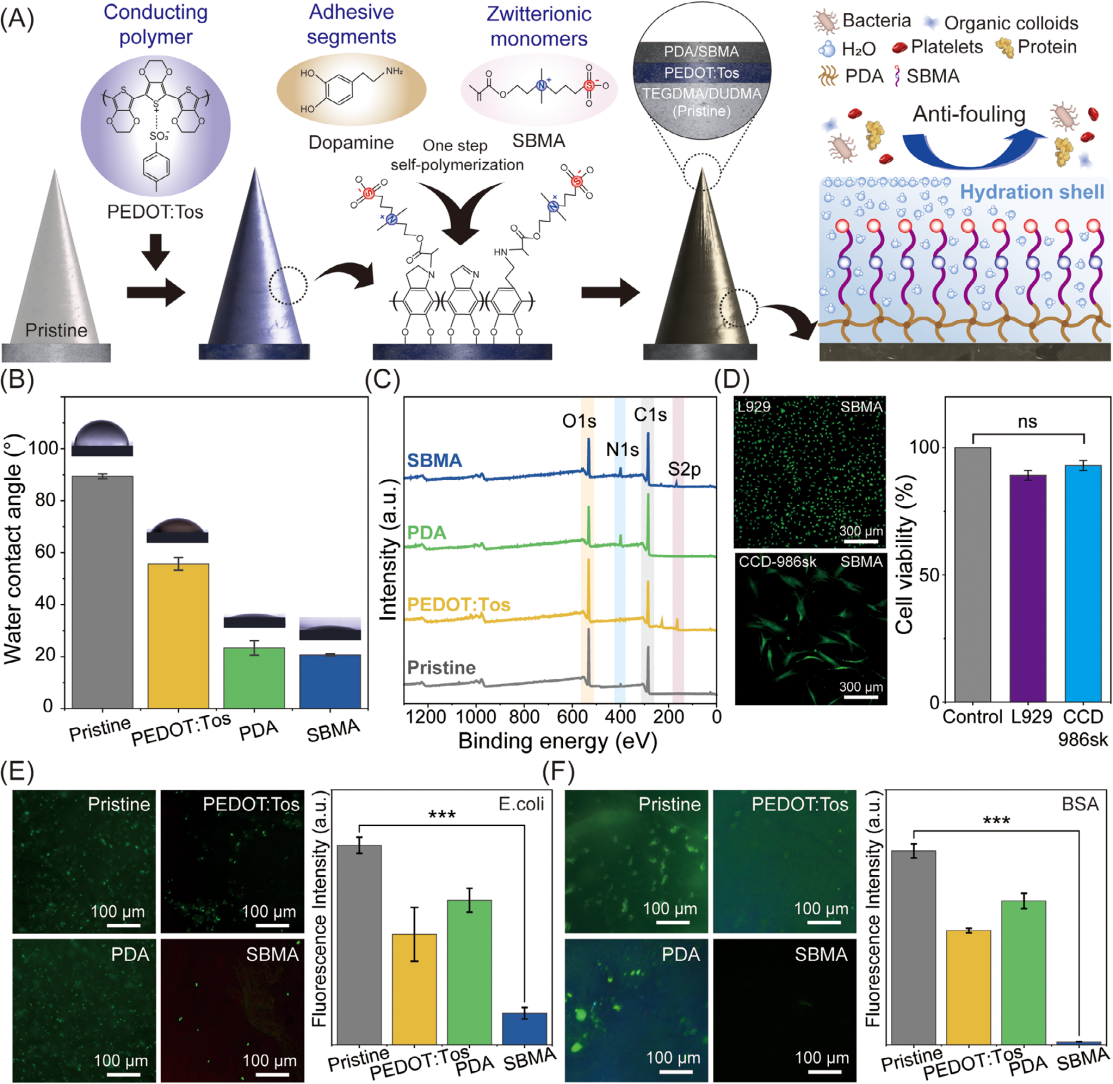
Antifouling and biocompatibility analysis of the SBMA‐coated MNE. A) Schematic of the workflow on the MNE involving surface modification of the zwitterionic SBMA for antifouling functionality. B) SBMA‐coated MNE demonstrated high hydrophilicity, with a contact angle of 20.65° ± 0.449°. Measurements were performed on five samples, with error bars indicating the standard deviations. C) Wide‐range XPS spectra indicating surface composition at different surface modifications: pristine, PEDOT:Tos‐coated, PDA‐coated, and SBMA‐coated MNEs. D) Cell viability (live/dead) assay and corresponding fluorescence intensity of L929 (mouse fibroblast cell line) and CCD‐986sk (human dermal fibroblast cell line) after 3 d of exposure to SBMA‐coated MNEs. Green fluorescence represents live cells, whereas red indicates dead cells. “ns” denotes no statistically significant difference. Antifouling performance of different surface modifications evaluated by E) bacterial adhesion of *E. coli* and F) protein adsorption using BSA‐FITC. All measurements were performed on five samples, with error bars indicating the standard deviations.

To inhibit biofilm formation on MNEs, a surface modification strategy based on the PDA‐SBMA copolymer coating was employed, as shown in **Figure** **2**A. The copolymerization was initiated by dopamine oxidation under alkaline conditions, generating dopamine quinone intermediates that underwent intramolecular cyclization, rearrangement, and subsequent polymerization to form PDA (Figure S1, Supporting Information).^[^ ^35^ ^]^ Subsequently, the zwitterionic monomer SBMA was introduced onto the PDA‐coated surface to form a PDA‐SBMA copolymer layer through two distinct conjugation pathways. The first pathway involved covalent conjugation of SBMA onto the PDA surface via Michael addition, which allowed stable immobilization of SBMA chains onto the PDA scaffold.^[^ ^36^ ^]^ Alternatively, SBMA reacted with oxidized catechol groups in PDA (dopamine quinone) through Schiff‐base chemistry, contributing to the formation of PDA‐SBMA copolymers. This hybrid coating exhibited strong adhesion to MNEs, attributed to the universal adhesive properties of PDA. In parallel, the zwitterionic moieties of SBMA formed a tightly bound hydration layer stabilized by hydrogen bonding, effectively preventing nonspecific biomolecular adsorption even under complex biological environments.

To evaluate changes in surface wettability, water contact angle (WCA) measurements were performed on MNEs before and after each modification step. As shown in Figure 2B, the WCA decreased from 89.44° ± 0.89° (pristine) to 55.71° ± 2.43° (PEDOT:Tos) and further to 20.65° ± 0.45° following SBMA coating, indicating a progressive increase in surface hydrophilicity. This enhancement is attributed to the introduction of densely packed hydrophilic chains that form strong interactions with interfacial water molecules.^[^ ^37^ ^]^

To confirm the surface modification, X‐ray photoelectron spectroscopy (XPS) analysis was conducted. The wide range of spectra (Figure 2C) clearly differentiated the elemental compositions of pristine, PEDOT:Tos, PDA‐, and SBMA‐modified MNEs. High‐resolution S 2p spectra exhibited characteristic doublets at 162.8 and 163.9 eV, corresponding to the thiophene ring of PEDOT, and an additional doublet at 167.4 and 169.1 eV assigned to the tosylate anion, validating successful PEDOT:Tos deposition (Figure S2, Supporting Information).^[^ ^38^ ^]^ Following the coating with PDA, the N 1s spectrum exhibited a pronounced peak at 399.2 eV, corresponding to the –NH_3_ group in PDA, which reflects the indole structure generated by dopamine self‐polymerization^[^ ^37^ ^]^ (Figure S3A, Supporting Information). In contrast, the high‐resolution N 1s spectrum of the SBMA‐coated MNEs revealed a distinct C–N⁺ peak at 402.1 eV, indicating the presence of quaternary ammonium groups derived from SBMA (Figure S3B, Supporting Information).^[^ ^39^ ^]^ Additionally, a weak signal observed at lower binding energies was attributed to the –SO_3−_ groups of SBMA, further supporting the coating of SBMA on the MNE surface.

The biocompatibility of the SBMA‐coated MNE was evaluated using L929 (mouse fibroblast) and CCD‐986sk (human dermal fibroblast) cell lines through both viability and cytotoxicity assays (Figure 2D). After 3 days of incubation with SBMA‐coated MNEs, the viability of L929 and CCD‐986sk cells remained comparable to that of the untreated control group, with values of 89.2% and 93.0%, respectively. Cytotoxicity was further assessed using a lactate dehydrogenase (LDH) assay, which measures membrane integrity according to enzyme leakage, in accordance with ISO 10993‐5 guidelines. The LDH release from cells exposed to SBMA‐coated MNE extracts remained low (5.3% and 3.9%, respectively), substantially below the cytotoxic threshold and significantly lower than that of the positive control (Figure S4, Supporting Information). These results indicate that the SBMA‐coated MNE induced minimal cellular damage and exhibited excellent biocompatibility.

Finally, the antifouling performance of the SBMA‐coated MNEs was evaluated against *Escherichia coli (E. coli)* and bovine serum albumin (BSA). As shown in Figure 2E, the bacterial attachment was significantly suppressed on SBMA‐coated MNEs compared with pristine, PEDOT:Tos‐coated, and PDA‐coated MNEs after exposure to an *E. coli* suspension. While the pristine surfaces exhibited dense biofilm formation with extensive bacterial aggregates, the SBMA‐coated MNEs showed minimal *E. coli* colonization, resulting in an 84.2% reduction in surface‐adhered cells. Furthermore, BSA adsorption was markedly reduced on SBMA‐coated MNEs, as evidenced by a 98.6% reduction in fluorescence intensity relative to pristine controls (p < 0.001, Figure 2F). These results demonstrate the strong antifouling capability of SBMA‐coated MNEs against both bacterial and protein fouling, which maintained interfacial integrity critical for reliable biosignal acquisition.

### 2.2 Skin Insertion Force

**Figure 3:**
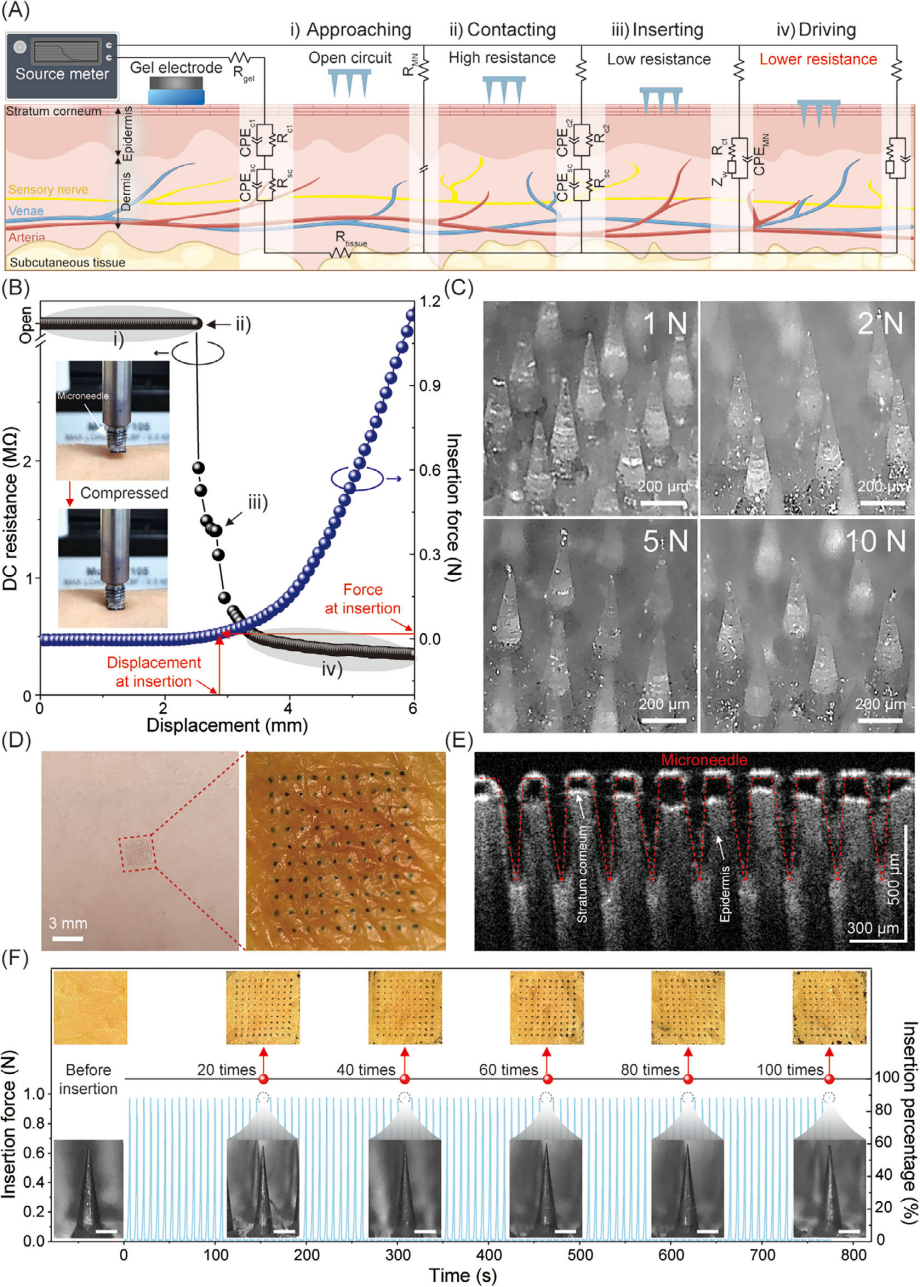
Skin insertion analysis of the MN array. A) Schematic of the MNE–skin equivalent circuit model. A gel electrode was placed on the skin as the counter electrode to establish a closed loop with the MNE. B) Plot of insertion force and DC resistance versus loading displacement during the insertion and removal of the MNE on human skin (inset: experimental setup). C) Optical images of the MNE after applying compressive forces ranging from 1 to 10 N. No fractures or fragment formation were observed, even at 10 N. D) Optical image of trypan blue‐stained skin showing distinct indentations from MN penetration, with no visible skin irritation after 6 h. E) OCT image captured immediately after inserting the MNE into the researcher's chest. The MNE could create pores and penetrate the skin insertion upon manual application. F) Insertion force and efficiency over 100 repeated applications on porcine skin. Insets: optical (top) and SEM (bottom) images indicating pierced porcine skin and MNE. Trypan blue staining was utilized to distinguish pierced from unpierced areas.

The mechanical strength of the SBMA‐coated MNEs was evaluated using a source meter and a motorized strain gauge to simultaneously measure insertion force and interfacial DC resistance during penetration. As shown in **Figure** **3**A, the insertion process was divided into four sequential stages—approaching (i), contacting (ii), inserting (iii), and driving (iv)—each corresponding to a distinct change in the electrical interface between the MNEs and skin. The equivalent circuit models displayed above each stage represent the progressive decrease in interfacial resistance as the MNE penetrates through the stratum corneum and reaches deeper tissue layers. Initially, high impedance was observed owing to the insulating nature of the skin surface. Upon penetration, the circuit transitioned to a lower‐resistance configuration, reflecting enhanced ionic contact with the viable epidermis. The electrical components and parameters of each model are detailed in Note S1 (Supporting Information).

These electrical responses were monitored in parallel with mechanical insertion measurements. As shown in Figure 3B, the applied force increased steadily until a sharp drop in DC resistance (green) signaled the breach of the stratum corneum, while the insertion force (sky blue) continued to rise during deeper advancement. The average insertion force was measured at 0.018 ± 0.0028 N, offering a twofold safety margin over the 0.058 N threshold previously reported for skin penetration using polymeric microneedles (MNs) of comparable geometry.^[^ ^40^ ^]^ The nearly linear force–displacement profile confirmed that the MNEs remained within the elastic deformation regime without structural failure.^[^ ^41^ ^]^

To further assess the mechanical robustness of the SBMA‐coated MNEs, compression testing was conducted under forces ranging from 1 to 10 N. As shown in Figure 3C, slight tip bending was observed at 5 N, but the MNEs remained structurally intact without significant deformation or fracture, even at the highest applied load. These results demonstrate that the MNEs possess sufficient mechanical strength for reliable insertion into the skin.

Following the evaluation of mechanical strength, the skin penetration capability of the SBMA‐coated MNE was assessed via trypan blue staining. A 10 × 10 MNE array loaded with 0.05 mg of trypan blue dye was manually applied to human skin. As shown in Figure 3D, optical images revealed a uniform pattern of blue‐stained dots corresponding to the MN layout, indicating successful and consistent skin insertion. To verify the penetration depth, optical coherence tomography (OCT) imaging was performed, which demonstrated the structural uniformity of the SBMA‐coated MNE prior to skin insertion (Figure S5, Supporting Information). As shown in Figure 3E, the high‐resolution cross‐sectional images clearly distinguished the skin layers, with the stratum corneum (10–20 µm) appearing as a thin, high‐intensity layer and the viable epidermis (75–150 µm) exhibiting a lower‐intensity response.^[^ ^42^ ^]^ The SBMA‐coated MNEs penetrated the stratum corneum, reaching an average depth of 361.17 µm (Figure S6 and Table S1, Supporting Information).

Finally, the insertion reliability of the SBMA‐coated MNE was evaluated through 100 consecutive insertion tests on porcine skin using the same device (Figure 3F). The insertion efficiency, calculated as the ratio of stained puncture sites to the number of holes, consistently exceeded 100% across all cycles, indicating uniform and complete skin penetration. Optical and scanning electron microscopy (SEM) imaging confirmed that the SBMA‐coated MNE tips retained their original geometry without visible fractures, cracks, or deformation.

### 2.3 Electrochemical Analyses

**Figure 4:**
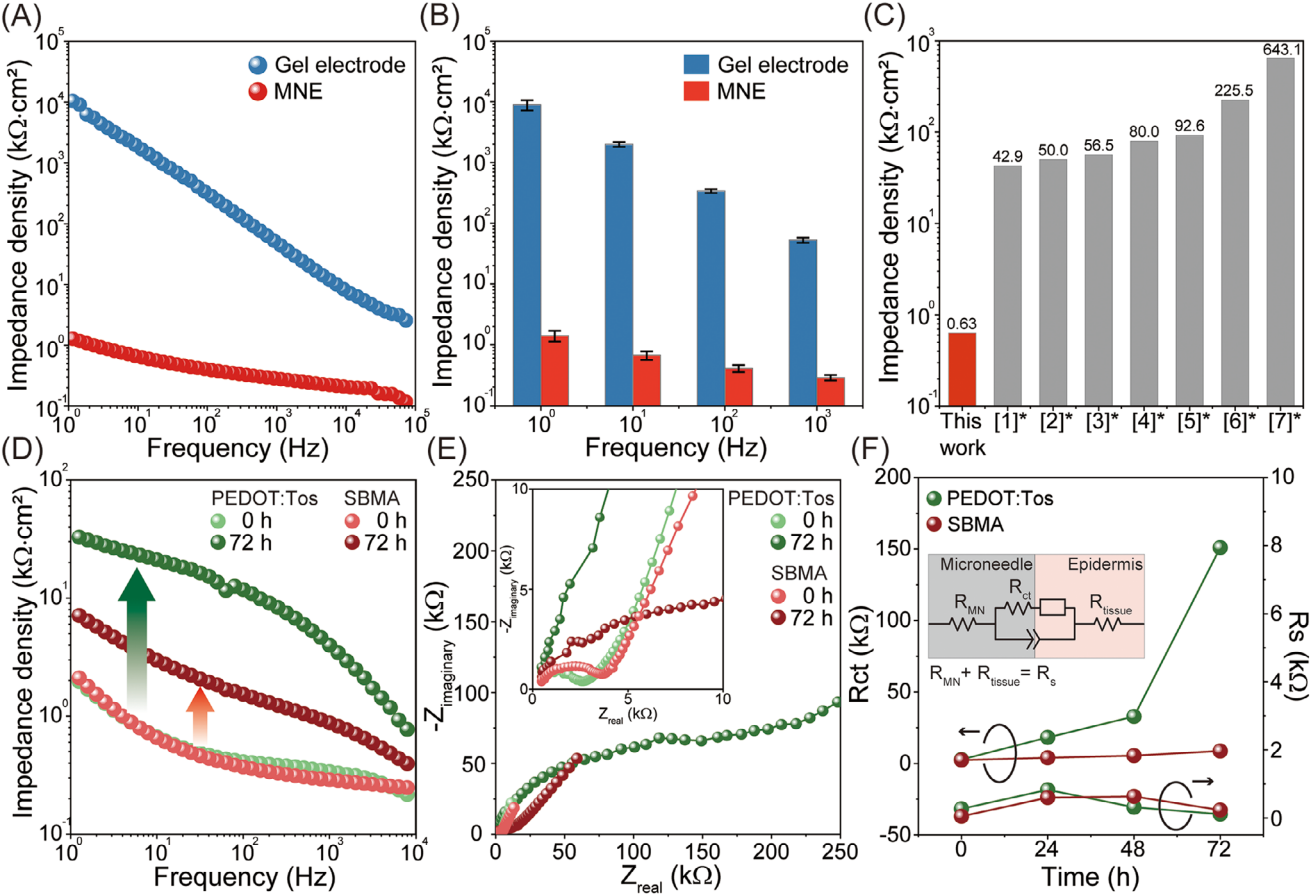
EIS results: A) electrode–skin interface impedance density spectra of the commercial gel electrode and MNE, and B) corresponding impedance densities at 1, 10, 100, and 1000 Hz, indicating that the SBMA‐coated MNE significantly reduces impedance compared with the gel electrode. C) Comparative overview of the impedance density across various reported ECG MNEs based on the data from Table S2 (Supporting Information). D) Time‐dependent impedance density spectra for PEDOT:Tos‐ and SBMA‐coated MNEs, validating the long‐term effectiveness of the antifouling coating. E) Nyquist plots indicating the real (Ζ′) and imaginary (Ζ′′) parts of the impedance of the MN–skin interface for PEDOT:Tos‐ and SBMA‐coated MNEs. All experimental results were subjected to a 10‐mV root‐mean‐square AC voltage. F) Changes in charge‐transfer resistance (*R_ct_*) and solution resistance (*R_s_*) from 0 to 72 h. An equivalent electrochemical circuit is shown in the inset.

To systematically evaluate the electrochemical characteristics of the SBMA‐coated MNE, EIS was performed to analyze the charge‐transfer behavior at the skin–electrode interface. The MNE was inserted into the chest of a human participant, and the impedance spectra were recorded across a wide frequency range. As shown in **Figure** **4**A,B, the SBMA‐coated MNE exhibited consistently lower impedance density than conventional gel electrodes, particularly in the low‐frequency range (<1 kHz), which is critical for acquiring high‐fidelity surface biopotential signals. Nyquist plot analysis (Figure S7, Supporting Information) supported this trend. The reduced semicircle diameter at high frequencies suggests a marked decrease in charge‐transfer resistance, likely due to enhanced Faradaic activity at the hydrated zwitterionic interface. This electrochemical behavior underscores the efficient ionic conduction and interfacial stability imparted by the SBMA coating. Importantly, the impedance density measured at 10 Hz was substantially lower than previously reported values for ECG‐compatible MNEs (e.g., 0.63 kΩ∙cm^2^), as indicated by Figure 4C and Table S2 (Supporting Information).

Next, we evaluated the long‐term impedance changes of PEDOT:Tos‐ and SBMA‐coated MNEs to examine the impact of biofilm formation on electrochemical performance. Biofilm accumulation on the surface of MNEs increases interfacial impedance, which can significantly compromise ECG quality by leading to baseline drift, noise, and morphological distortion in the recorded waveforms.^[^ ^43^, ^44^ ^]^ As shown in Figure 4D, the SBMA‐coated MNE exhibited only a slight increase in impedance density at 10 Hz—from 0.60 to 2.7 kΩ∙cm^2^ over a 72‐h period. In contrast, the PEDOT:Tos‐coated MNE showed a pronounced rise from 0.63 to 19.0 kΩ∙cm^2^ under the same period.

Figure 4E presents Nyquist plots that track the time‐dependent changes in interfacial charge‐transfer behavior. Initially, the PEDOT:Tos‐ and SBMA‐coated MNEs demonstrated similar impedance characteristics across the full frequency range, indicating comparable initial interfacial behavior. However, distinct electrochemical differences emerged with time in the semicircles and slopes of the Nyquist plots in the low‐frequency region, reflecting evolving interfacial interactions related to surface fouling at the MNE–skin interface.

To quantify these changes, the charge‐transfer resistance (Rct) and solution resistance (Rs) were extracted via equivalent circuit fitting, as shown in Figure 4F. The inset depicts the Randles equivalent circuit, in which Rs denotes the solution resistance and Rct represents the charge‐transfer resistance at the electrode–tissue interface. Over 72 h, Rs remained stable for both coatings, indicating no observable mechanical failure such as delamination or cracking of the conductive layer.^[^ ^45^, ^46^ ^]^ In contrast, the Rct of the PEDOT:Tos‐coated MNE markedly increased from 2.78 to 150.99 kΩ, suggesting a substantial loss of electrochemically active surface area due to interfacial fouling.

In parallel, the Warburg diffusion resistance (Zw) also exhibited a significant increase, attributed to a rise in the Warburg coefficient (σ), as shown in Figure S8 (Supporting Information). The elevated Zw indicates hindered ion transport at the MNE–skin interface, likely resulting from the formation of longer ion‐diffusion diffusion pathways during electrochemical cycling. These findings point to the development of a non‐conductive biofilm at the interface, which impairs Faradaic charge transfer and ion mobility, increasing both Rct and Zw. This fouling phenomenon is primarily driven by nonspecific adsorption of proteins and other biomolecular residues, which compromise the electrochemical performance of the electrode over time.^[^ ^47^, ^48^, ^49^ ^]^

### 2.4 Long‐Term ECG Signal Monitoring

**Figure 5:**
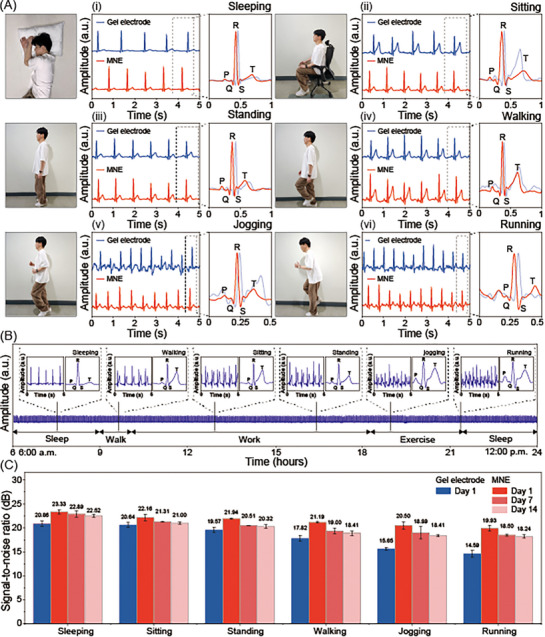
Comparison of real‐time long‐term ECG data and SNR between the commercial gel and MNEs. A) Photographs of different motions and the corresponding ECG signals recorded using a wearable wireless system during (i) sleeping, (ii) sitting, (iii) standing, (iv) walking (≈3 km h^−1^), (v) jogging (≈5 km h^−1^), and (vi) running (≈7 km h^−1^) measured using a gel electrode (blue) and MNEs (red). Data was collected immediately after the electrode attachment. B) Real‐time 24‐h ECG data obtained after 14 d of continuous MNE wear, along with representative ECG signals for each gesture. C) Quantitative analysis of the calculated SNR of ECG signals recorded using the gel electrode and MNEs after 1, 7, and 14 days. To obtain long‐term data, the MNE was worn continuously on the researcher's chest for 14 days.

To evaluate the long‐term stability of the SBMA‐coated MNEs under real‐world conditions, we assessed its ability to monitor ECG signals during various daily physical activities using a wearable wireless system. The SBMA‐coated MNEs and a commercial gel electrode were placed in close proximity on the left side of the chest. As shown in **Figure** **5**A, ECG recordings were collected during representative activities, including sleeping (i), sitting (ii), standing (iii), walking (iv), jogging (v), and running (vi).

During static motions, such as sleeping, sitting, and standing, both the SBMA‐coated MNE and gel electrode produced stable and comparable ECG signals, without baseline drift or waveform distortion. In contrast, during dynamic motion, including walking, jogging, and running, the gel electrode exhibited pronounced signal degradation, characterized by baseline instability and waveform fluctuation (Figure S9A, Supporting Information). The SBMA‐coated MNEs, however, consistently preserved signal integrity with minimal noise and clear waveform morphology, even under vigorous motion (Figure S9B, Supporting Information).

Given the critical importance of maintaining reliable ECG monitoring in daily life, we further evaluated the long‐term signal quality of the SBMA‐coated MNEs during routine activities. Continuous ECG recordings were obtained over a 14‐day period on days 1, 7, and 14, from 6:00 a.m. to 12:00 p.m., while the volunteer engaged in typical activities such as sleeping, working, walking, and exercising (Figure 5B; Figures S10 and S11, Supporting Information). The expanded waveform segments in the insets clearly reveal P‐waves, QRS complexes, and T‐waves, underscoring the ability of the SBMA‐coated MNE to capture high‐fidelity biopotential signals. We assessed the quality of ECG signals acquired during physical activity by quantifying the SNR and standard deviation of noise (STD) using MATLAB's advanced signal‐processing framework (Figure 5C; Figure S12, Supporting Information). The SNR serves as a key metric for evaluating signal clarity and recording fidelity in MN‐based systems. Notably, the SBMA‐coated MNEs exhibited only a slight reduction in SNR during high‐intensity activity (e.g., running), decreasing from 23.33 to 19.93 dB over the 14‐day period. In contrast, gel electrodes showed a larger reduction—from 20.86 to 14.59 dB—under similar conditions. Remarkably, the SBMA‐coated MNE maintained higher SNR values on day 14 compared with the initial readings obtained from the gel electrodes.

Finally, we evaluated the skin condition after removal of the MNEs following 7 and 14 days of continuous wear. As shown in Figure S13 (Supporting Information), only mild erythema was observed at the MNE sites, without signs of edema, abrasion, or persistent irritation. These marks were transient and subsided within 1 h, indicating excellent skin compatibility of the SBMA‐coated MNEs. In addition, digital microscopy images of the MNEs confirmed that they maintained their structural integrity, without tip deformation, detachment, or fracture during or after use. Collectively, these findings underscore the potential of SBMA‐coated MNEs for robust, long‐term ECG monitoring in dynamic, real‐world environments.

### 2.5 Limitations

Despite the promising performance of the SBMA‐coated all‐polymeric MNEs, several limitations of the present study should be acknowledged. First, the in vivo validation was conducted on three healthy volunteers, which may not fully represent the physiological and dermatological variability observed in broader populations, including differences in skin thickness, stratum corneum integrity, hydration level, sebaceous secretion, pigmentation, and local sweat gland density that can affect the electrical and mechanical interaction between the MNEs and the skin. Second, the fabrication of MNEs was conducted at the laboratory scale using a 2PP‐based master template, followed by polydimethylsiloxane (PDMS) replication and polymer casting. While this approach ensures high‐resolution prototyping, the throughput and scalability remain constrained compared with industrial manufacturing. Nevertheless, the process is readily transferable to scalable techniques such as photolithography or deep reactive‐ion etching for large‐area master mold fabrication, enabling wafer‐ or panel‐scale replication. The subsequent UV polymerization and surface functionalization steps—PEDOT:Tos deposition and zwitterionic SBMA coating—are also compatible with automated plasma treatment and continuous coating methods widely used in biomedical device production, suggesting a clear pathway toward large‐scale manufacturing.

To overcome these limitations, future work will focus on (i) expanding human trials across diverse skin types and demographic groups, (ii) optimizing the fabrication process toward high‐throughput and automated manufacturing, thereby bridging the gap between laboratory‐scale prototyping and practical applications.

## 3 Conclusion

We developed a fully polymeric, biocompatible MNEs with high mechanical integrity and ultra‐low interfacial impedance, optimized for long‐term ECG monitoring under real‐world motion conditions. These MNEs are designed for integration into wearable, wireless systems for continuous ECG monitoring. The fabrication process was optimized to produce sharp, well‐defined MNEs that allow reliable penetration, ensuring stable electrode–skin contact. For improving both electrical conductivity and biofouling resistance, the MNE surface was sequentially coated with PEDOT:Tos to reduce interfacial impedance, followed by zwitterionic SBMA to prevent nonspecific protein adsorption and cellular adhesion.

This surface modification strategy enhanced the electrochemical characteristics of the electrode while providing long‐term antifouling properties. In‐depth EIS analyses demonstrated that the SBMA coating effectively resisted biofouling and maintained a stable impedance over extended use. Notably, the SBMA‐coated MNEs achieved a low impedance of 0.63 kΩ·cm^2^ at 10 Hz, demonstrating their suitability for long‐term, high‐fidelity ECG acquisition in wearable monitoring systems. Furthermore, signal quality assessments based on STD and SNR measurements on days 1, 7, and 14 confirmed that the MNEs consistently maintained stable ECG recordings over time, demonstrating their reliability for both short‐ and long‐term monitoring. Collectively, these findings demonstrate the potential of SBMA‐functionalized polymeric MNEs as a stable and reliable platform for continuous, long‐term electrophysiological monitoring, positioning them as promising candidates for next‐generation wearable health diagnostics and human–machine interface technologies.

## 4 Experimental Section

### Materials

DUDMA, TEGDMA, phenylbis(2, 4, 6‐trimethylbenzoyl)phosphine oxide (photoinitiator), 3,4‐ethylenedioxythiophene 97% (EDOT), trichloro(1H,1H,2H,2H‐perfluorooctyl)silane, iron(III) p‐toluene sulfonate, pyridine, dopamine hydrochloride, [2‐(methacryloyloxy)ethyl]dimethyl‐(3‐sulfopropyl)ammonium hydroxide (SBMA), 2‐amino‐2‐(hydroxymethyl)‐1,3‐propanediol (Tris base), and trypan blue were purchased from Sigma‐Aldrich (St. Louis, MO, USA) and utilized without further purification. Polydimethylsiloxane (PDMS) pre‐polymer (Sylgard 184A) and a curing agent (Sylgard 184 B) were purchased from Dow Corning Co., Ltd. (Shanghai, China). Deionized (DI) water (over 18 MΩ cm, 25 °C) was utilized throughout the experiment. The 2223H Ag/AgCl gel electrode was purchased from 3 M (Saint Paul, MN, USA), isopropyl alcohol (IPA) was purchased from Samchun Chemical Co. (Seoul, Korea), and isomalt was commercially acquired.

### Fabrication of MNEs

The stepwise fabrication process for MNEs is shown in Figure S14 (Supporting Information). The process commenced with the fabrication of a conical MN master structure, featuring a root diameter and height of 150 and 450 µm, respectively, precisely patterned using a two‐photon polymerization 3D printer (Nanoscribe, Germany). To generate a negative mold, a mixture of pre‐polymer and curing agent in a 10:1 ratio was poured over the MN master and thermally cured at 60 °C for 1 h, resulting in a flexible PDMS negative master mold. This approach ensured high‐fidelity replication of the MN geometry, laying the groundwork for subsequent fabrication steps. The PDMS negative master mold underwent surface modification to enhance mold release and replication fidelity. Initially, it was subjected to oxygen plasma (CUTE, Femtoscience, South Korea) treatment at 30 W for 1 min, followed by exposure to trichloro(1H,1H,2H,2H‐perfluorooctyl)silane in a vacuum oven at 60 °C for 24 h. This process resulted in the formation of a superhydrophobic self‐assembled monolayer (SAM) on the PDMS surface, reducing adhesion during subsequent fabrication steps. Using the SAM‐coated PDMS mold, PDMS MNs were fabricated by molding an isomalt aqueous solution under vacuum at 150 °C, ensuring precise replication of the MN geometry. Once the sugar template solidified, a precursor solution composed of DUDMA and TEGDMA in a 2:8 ratio with 0.5 wt% photoinitiator was introduced into the mold under a vacuum of 0.03 Torr to guarantee complete infiltration of the microstructure. UV curing (Model 18, Jetlight Company) was performed for 5 min, which provided sufficient radical propagation to achieve uniform conical geometries with sharp tips (Figure S15, Supporting Information). Finally, precisely structured MNs were obtained by dissolving the sugar template in water.

### Surface Modification

A PEDOT:Tos coating was applied to form a conductive surface on the MNEs. The precursor solution was prepared by dissolving iron(III) p‐toluenesulfonate in IPA at a concentration of 10 wt%, followed by the addition of 30 µL of EDOT per 1 mL of solution. The conductive layer was formed through a dip‐coating process in which the MNs were sequentially immersed in the PEDOT:Tos precursor solution. An optimal conductive layer was established for four coating cycles after evaluating the impedance across multiple coating cycles (Figures S16 and S17, Supporting Information). To introduce antifouling properties, the PEDOT:Tos‐coated MNEs underwent further surface modifications. First, the electrodes were subjected to oxygen plasma treatment (60 W for 1 min) to enhance surface reactivity. Subsequently, the MNEs were immersed in a Tris base solution (pH 8.5) containing 2 mg mL^−1^ dopamine hydrochloride and SBMA at a concentration 60 times that of dopamine, as shown in Figure S18 (Supporting Information). The structural integrity and surface morphology of the modified MNEs were characterized using optical microscopy and SEM, as shown in Figure S19 (Supporting Information).

### Surface Characterization

XPS measurements were performed using a Nexsa‐G2 instrument (Thermo Scientific) equipped with a monochromatic Al X‐ray source (hν = 1486.6 eV). The system operated at 12 kV and 10 mA with a spot size of 400 µm. To assess the surface coating characteristics of the pristine resin, PEDOT:Tos‐coated, PDA‐coated, and SBMA‐coated MNs, WCA measurements were performed. WCA analysis was conducted by dispensing DI water onto various chemical surfaces and capturing side‐view photographs of the contact angle using a Phoenix‐MT digital camera. The images were then processed using ImageJ software.

### Electrode–Skin Impedance Evaluation

The electrode–skin impedance was measured using a two‐electrode system equipped with an electrochemical workstation (Interface 1010E, Gamry Instruments). Measurements were conducted over the frequency range of 10^0^ to 10⁵ Hz, with a 10‐mV AC perturbation. The corresponding current response at each frequency was simultaneously recorded by the electrochemical workstation as well as impedance spectra were obtained from the ratio of applied voltage to the measured current. Commercial Ag/AgCl gel electrodes and MNEs were attached to the center of a volunteer's chest for testing. The surface area of each Ag/AgCl gel electrode was 2.25 cm^2^, based on the dimensions of the gel portion, with the surface area of the MNEs estimated as 0.09 cm^2^.

The Warburg coefficient was calculated from the slope of the plot between the inverse square root of the frequency and Warburg diffusion resistance (Equation 1). The solution and charge‐transfer resistances were calculated using Equation 2.

(1) $$\sigma \propto \frac{Z_{w}}{\sqrt{w}}$$

(2) $$Z_{w}=Z_{real}-R_{s}-R_{ct}$$

here *σ*, *w*, and *Z_w_* denote the Warburg coefficient, frequency, and Warburg diffusion resistance, respectively. *Z_w_* is defined as the real‐axis impedance after subtracting the solution resistance and charge‐transfer resistance.

### Insertion Testing of MNEs

A hexahedral structure was installed on the head of the motorized strain gauge (MARK‐10, ESM 303) to ensure that the measurements were not influenced by undesirable contact with the skin. The MN was attached to the conical structure using epoxy. A copper wire, connected to a source meter (Model 2400, Keithley), was linked with the MNs using silver paste to establish a conductive path. A commercial Ag/AgCl gel electrode was attached to the back of the participant's hand and connected to the source meter, creating a closed loop upon contact of the MNs with the skin. The detailed setup is shown in Figure S20 (Supporting Information), with the insertion speed set at a constant velocity of 20 mm min^−1^. To assess the mechanical durability of the MNEs during repeated use, they were coated with trypan blue diluted in DI water using a fine‐tip paintbrush (Zem Brush MFG, OH, USA). Optical images of the MNEs were captured every 20 insertions immediately after skin insertion (prior to cleaning) to monitor potential MNE damage or coating degradation over time.

For further skin insertion testing, an OCT system was utilized to visualize a cross‐section displaying the penetration depth of the MNE. The electrode was inserted into the forehead of a healthy male participant using a simple launch method. A commercial spectral‐domain OCT system (HOCT‐1F, Huvitz, Inc.) designed for ophthalmology clinics, which utilized a superluminescent diode with a central wavelength of 840 nm, provided a lateral resolution of 20 µm and an axial resolution of 7 µm, which were suitable for imaging the operation of the MNEs. Because the default OCT imaging equipment was optimized for imaging the posterior chamber of the retina, an anterior segment adapter was utilized to capture skin images.

### In Vitro Biocompatibility Assay

To evaluate the biocompatibility of the fabricated microneedles, an in vitro cytotoxicity assay was conducted according to ISO 10993‐5 guidelines. Two fibroblast cell lines were used: L929 murine fibroblasts (RRID: CVCL_0462), a standard cell line for cytotoxicity screening, and CCD‐986sk human dermal fibroblasts (RRID: CVCL_2400), which better reflect the microneedle's potential interaction with human skin tissue (Korean Cell Line Bank, Korea). In summary, 2 × 10^4^ cells well^−1^ were cultured in a 24‐well plate for 24 h, after which each 0.5‐mm^2^ MN array was placed on top of the cells for up to 5 d (Figure S21, Supporting Information). To assess the cell viability, the cells were incubated with ethidium homodimer 1 (4 µM) and calcein‐AM (2 µM) for 30 min at 37 °C in a 5% CO_2_ (ThermoFisher, USA). After the cells were washed twice with PBS, they were subjected to fluorescence staining and examined using a confocal microscope (Zeiss, Germany). The fluorescence intensity was measured using a spectrophotometer (BioTek, USA). Additionally, the WST‐8 test was utilized to assess cell viability (WelGene, Korea). Cells incubated with MNE were incubated with 10 µL of the WST‐8 labeling solution for 1 h at 37 °C. The absorptance was measured at 450 nm using a spectrophotometer (BioTek). To assess the cytotoxicity of MNE, LDH released from damaged cells was measured using an LDH detection kit (DoGen Bio, Korea). Following exposure to MNE, the supernatants were collected for LDH measurement, and the absorbance of the medium was measured at 450 nm using a spectrophotometer (BioTek).

### Real‐Time ECG Monitoring System Design

The ECG monitoring system can be categorized into three components: (1) an ECG‐monitoring printed circuit board (PCB), (2) a battery pack, and (3) an MNE. The ECG PCB, as shown in Figure S22 (Supporting Information), incorporates an analog front‐end IC based on ADS1292 (18‐bit ADC) and a Bluetooth module (PLE‐52 with an antenna). The overall system architecture is shown in Figure S23 (Supporting Information). The battery pack comprised a 3.7‐V, 220‐mAh lithium‐polymer battery and TP4056‐based battery charging module bonded together using very high bonding tape. To ensure 24 h real‐time ECG monitoring, the battery charge/discharge test capacity was measured (Figure S24, Supporting Information). The ECG‐monitoring PCB and battery pack were enclosed within a 3D‐printed polylactic acid cover and bottom plate.

### System Integration with MNEs

The assembled device was mounted onto a flexible medical tape via an elastomer layer, forming a multilayer structure of the device, an elastomer, and a medical tape. Electrical connections between the microneedles and the device were established using 0.1‐mm‐diameter enamel‐coated copper jumper wires (Figure S25, Supporting Information). The enamel coating at the wire ends was removed via high‐temperature treatment to expose the conductive core. On the PCB side, each jumper wire was soldered to ensure robust electrical and mechanical fixation. The wires were then passed through a small hole in the PLA bottom plate and connected to the MNEs. On the MNE side, the exposed wire tip was positioned at the center of the backside of the MNE base and secured with silver paste to achieve reliable electrical contact. The connection was further reinforced with a medical tape to ensure mechanical stability and long‐term adhesion (Figure S26, Supporting Information).

### ECG Recording and Signal Analysis

ECG signals were recorded from three healthy male volunteers (age: 40 ± 3 years; height: 177.3 ± 5.4 cm; weight: 76.3 ± 7.2 kg) using both the MNEs and commercial gel electrodes. A wearable ECG monitoring system was employed for continuous data acquisition during various daily activities. Both types of electrodes were placed on the chest, with one positioned between V1 and V2 and the other inferior to V4, and secured using medical‐grade adhesive tape to ensure consistent skin–electrode contact throughout the recording period. A metronome was used to perform dynamic tests at specified speeds. The “walking” status involved dynamic walking at 80 steps min^−1^, the “jogging” state required subjects to move at 120 steps min^−1^ with a short step length, and the “running” state required subjects to move at 140 steps min^−1^ with a long step length.

To preprocess the raw ECG signals, a 0.5 Hz high‐pass digital filter was first applied to remove low‐frequency motion artifacts. A 59–61 Hz band‐stop filter was then employed to eliminate power‐line interference. Finally, a 20 Hz low‐pass digital filter was used to suppress high‐frequency noise while preserving the morphological characteristics of the ECG waveform.

The SNRs were calculated as follows:

(3) $$\mathrm{SNR}\left(\mathrm{DB}\right)=20\log_{10}\left(\frac{Peak}{STD}\right)$$

where “Peak” and “STD” denote the peak amplitude and standard deviation of the noise current, respectively.

## Conflict of Interest

The authors declare no conflict of interest.

## Author Contributions

J.H.K. and C.H. contributed equally to this work. J.H.K. and C.H. designed the experiments. J.H.K., C.H., J.H.L., and H.C.J. performed the experiments and collated the data. J.H.K., C.H., and J.H.L. analyzed the data. C.H. wrote the original draft. D.Y.K. performed data analysis and interpretation, conducted project administration, and reviewed the manuscript.

## Supporting information



Supporting Information

## Data Availability

The data that support the findings of this study are available from the corresponding author upon reasonable request.
