# Supplementary material for: Antifouling All‐Polymeric Microneedle Array for Long‐Term Wearable ECG Monitoring
Source: Adv Sci (Weinh). 2025 Nov 14;13(6):e12430. doi: 10.1002/advs.202512430 (PMC12866678; doi:10.1002/advs.202512430)
Supplement: Supplementary file 1 — Supporting Information [file ADVS-13-e12430-s001.docx]

Supporting Information

**Antifouling All-Polymeric Microneedle Array**

**for Long-Term Wearable ECG Monitoring**

*Ju Hyeon Kim*^†^*, Chuljin Hwang*^†^*, Jee Hoon Lee, Hang Chan Jo, and Dae Yu Kim**

J. K.

Department of Mechanical Engineering, Inha University, Incheon, 22212, Republic of Korea

C. H., H. J., D. K.

Department of Electrical and Computer Engineering, Inha University, Incheon 22212, Republic of Korea

J. L., D. K.

Inha Research Institute for Aerospace Medicine, Inha University, Incheon 22212, Republic of Korea

D. K.

Center for Sensor Systems, Inha University, Incheon 22212, Republic of Korea

*Corresponding Author: Prof. Dae Yu Kim (dyukim@inha.ac.kr)

**Supporting Information text**

The components explanation in equivalent circuit model.

R_gel_ and R_MN_ denote the electrical resistance between the source meter and the gel or microneedle (MN) electrodes, including resistance through the connecting wires. CPE_c1_ and CPE_c2_ represent constant phase elements that model the non-ideal capacitive behavior at the interface between the gel or MN electrodes and the stratum corneum (SC). The corresponding contact resistances at these interfaces are denoted as R_c1_ and R_c2_. R_sc_ and CPE_sc_ describe the intrinsic electrical resistance and capacitive characteristics of the SC layer. The charge transfer resistance and associated constant phase element between the MN electrode and the interstitial fluid (ISF) are represented by R_ct_ and CPE_MN,_ respectively. Z_w_ models the Warburg impedance, accounting for diffusion-related impedance between the MN and ISF. R_tissue_ represents the ionic resistance of the underlying subcutaneous tissue.

**
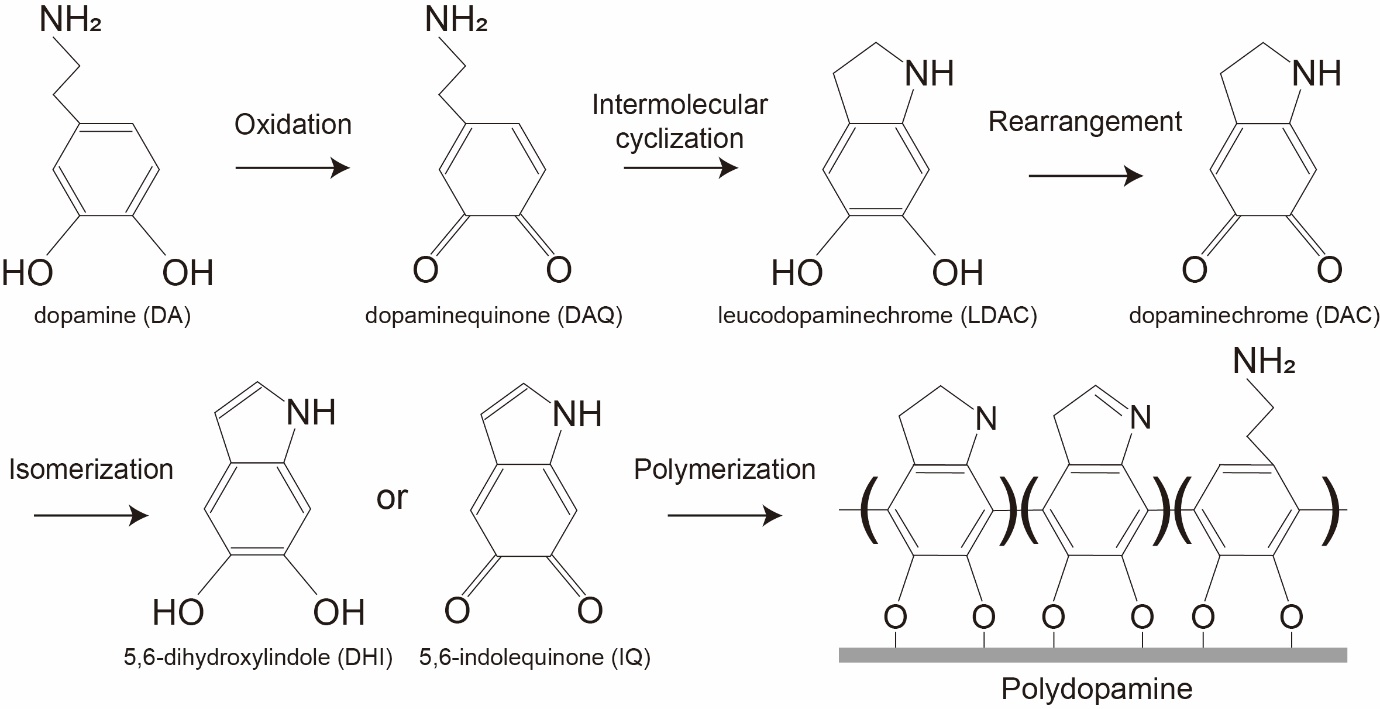
**

**Figure S1.** Suggested mechanism of dopamine polymerization.

**
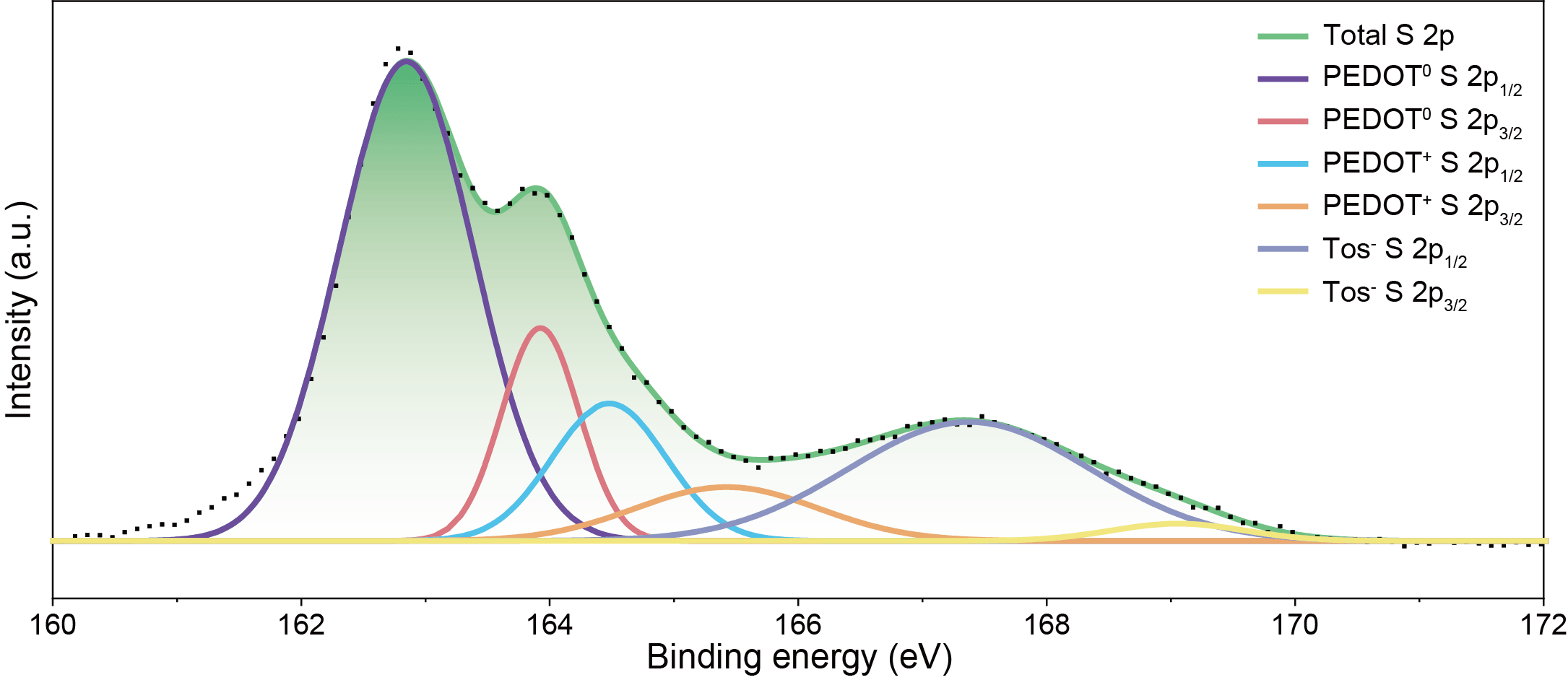
**

**Figure S2.** S 2p XPS data analysis of PEDOT:Tos. PEDOT:Tos has two different sulfur atoms within thiophene ring of PEDOT (161 to 166 eV) and tosylate ion (166 to 170 eV).


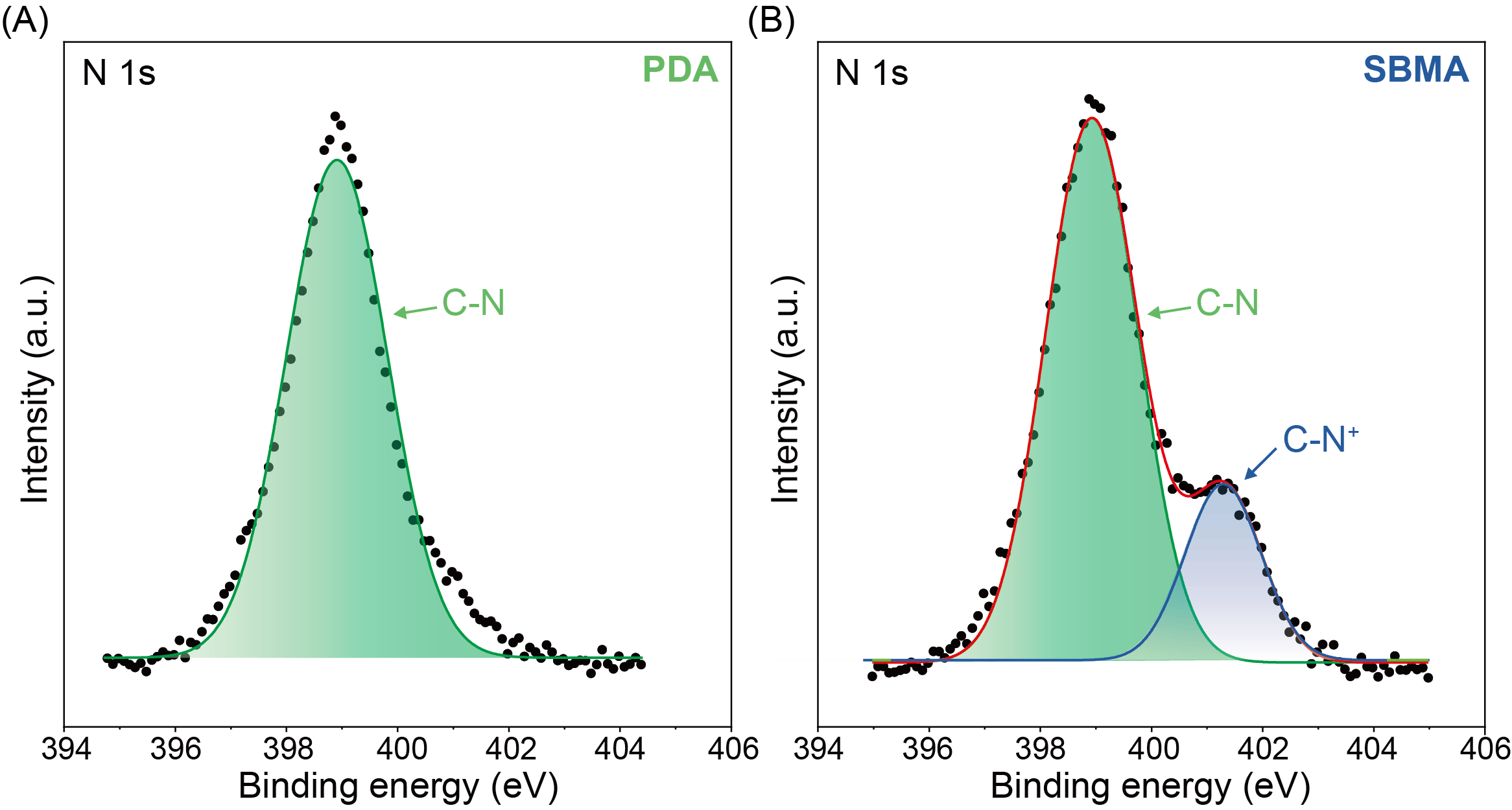


**Figure S3.** N 1s XPS data analysis of (A) PDA and (B) SBMA. SBMA has an additional C-N^+^ functional group (402.1 eV).

**
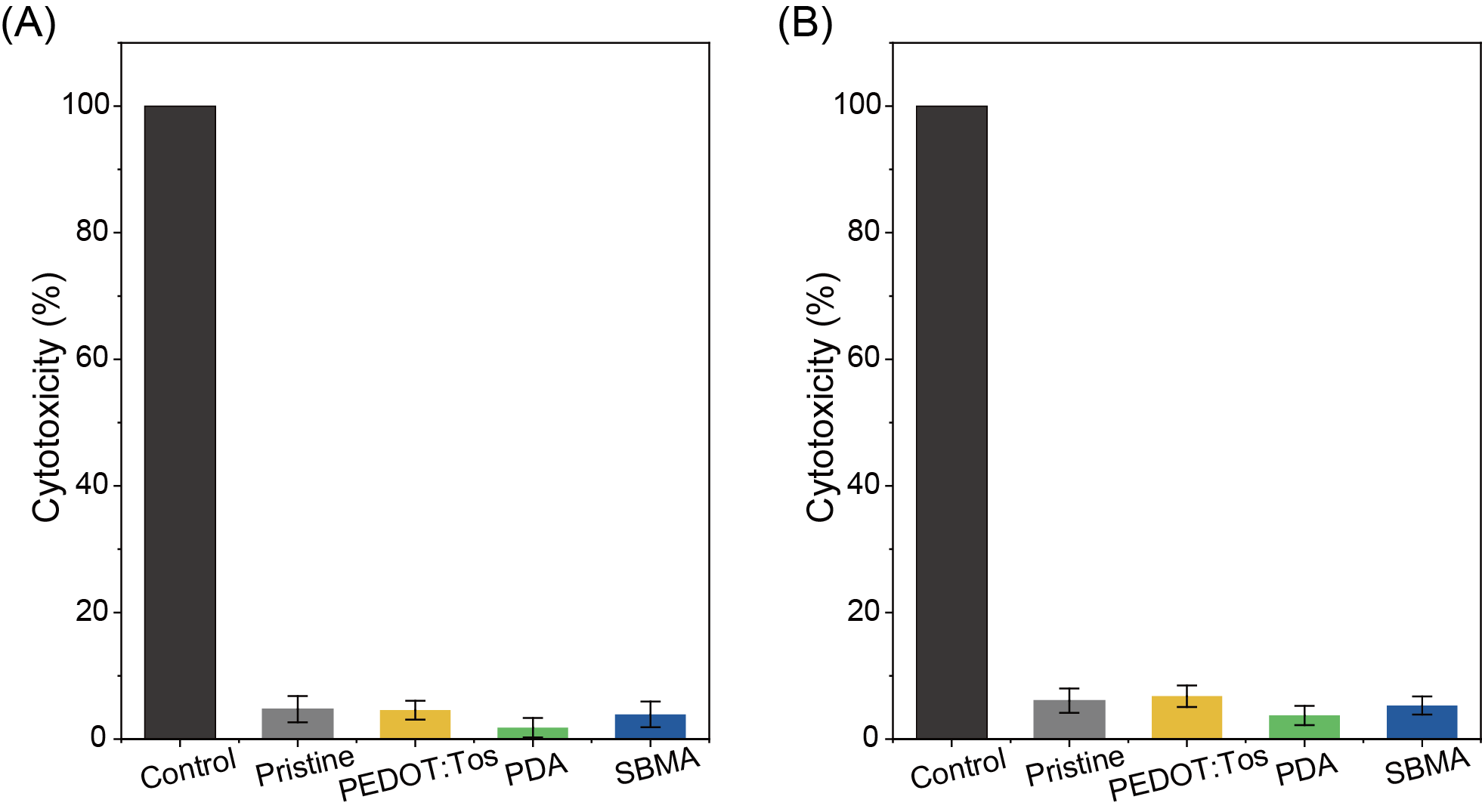
**

**Figure S4.** Cytotoxicity of SBMA-coated MNEs compared to the positive control group using (A) L929 (mouse fibroblast cell line) and (B) CCD-986sk (human skin fibroblast cell line).


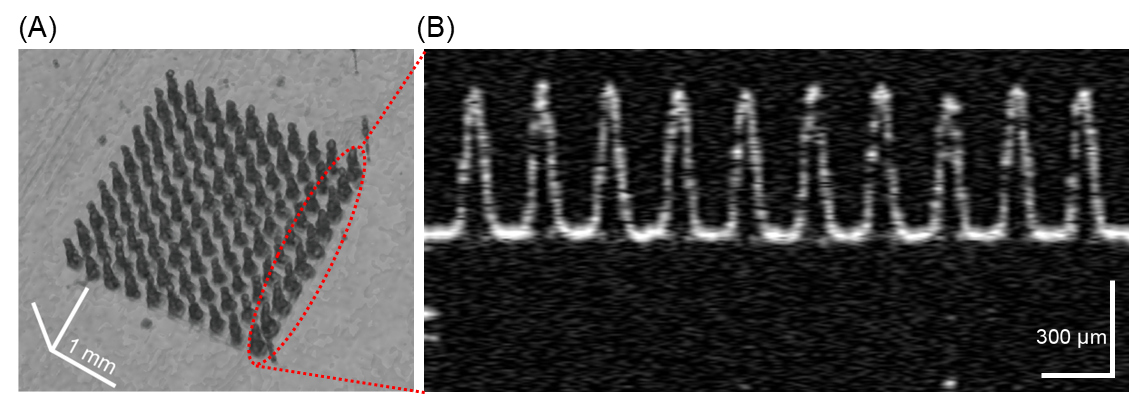


**Figure S5.** OCT image of (A) the fabricated SBMA-coated MNE array prior to skin insertion and (B) corresponding cross-sectional image.


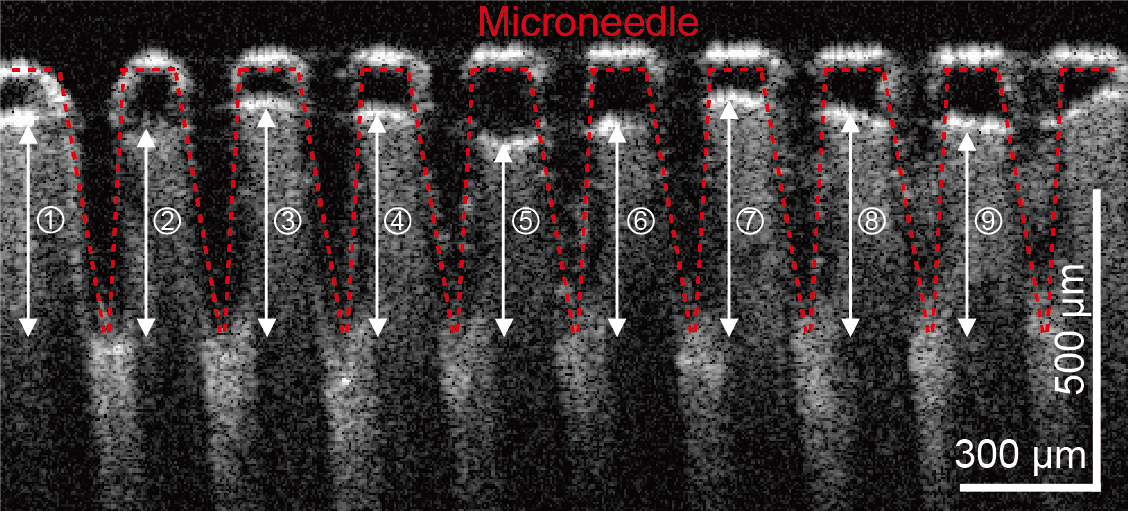


**Figure S6.** The OCT image captured immediately after inserting the MNE into the volunteer chest. The red dashed outlines indicate the geometry of each MNE, and the white arrows represent the measured penetration depths (①–⑨).


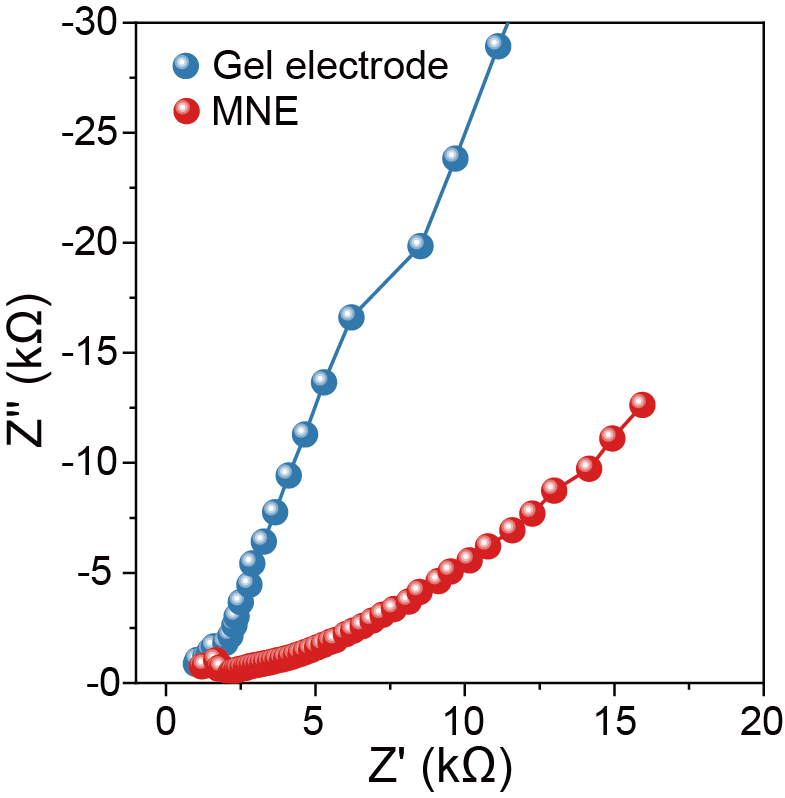


**Figure S7.** Nyquist plots of the gel electrode and SBMA-coated MNE. The gel electrode exhibits a longer, more vertical tail compared to the MNE, indicating higher capacitive behavior and diffusion impedance.

**
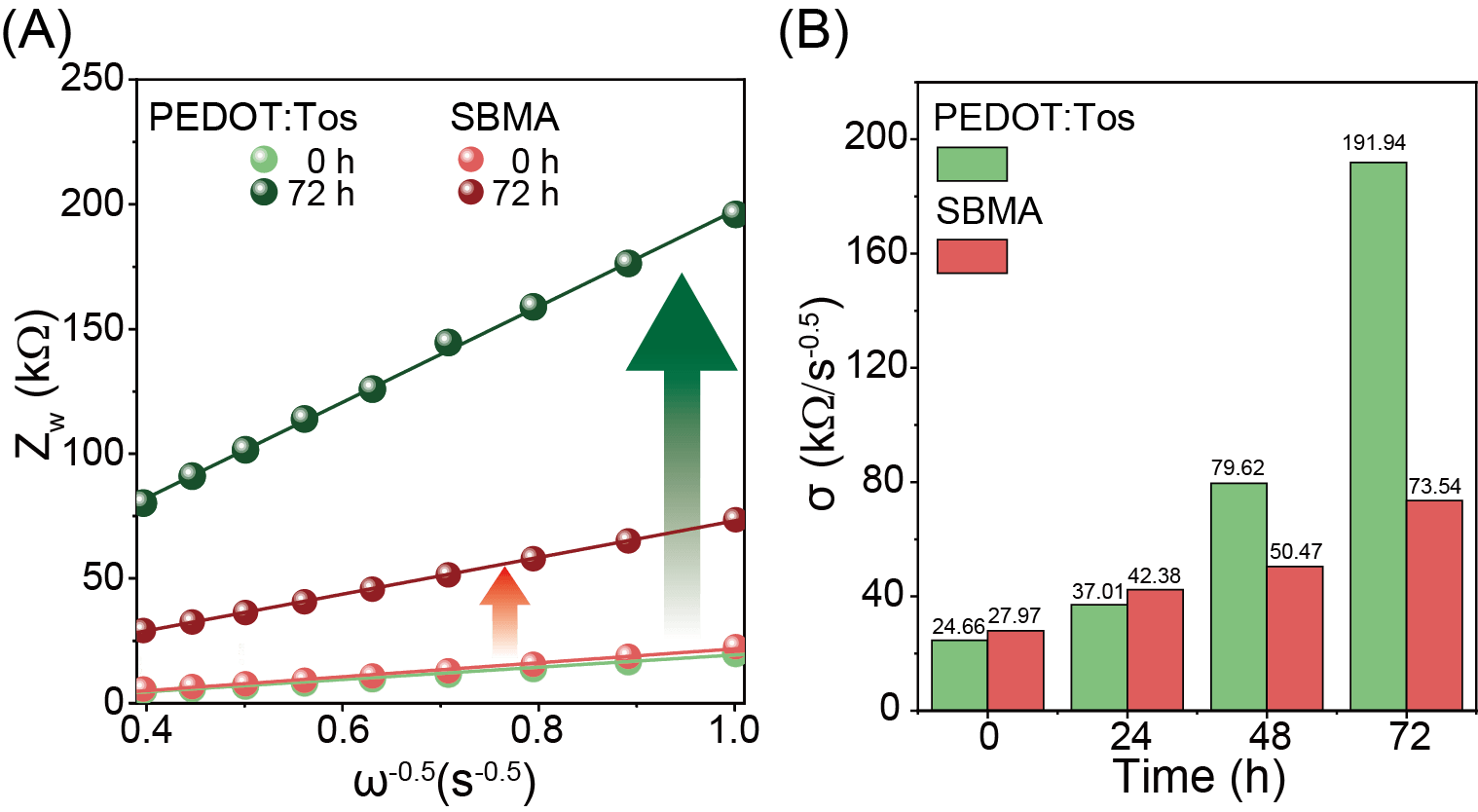
**

**Figure S8.** (A) Warburg diffusion resistance linear fitting about inversed root square of frequency and (B) the representation of the Warburg coefficient with PEDOT:Tos and SBMA coating at 0, 24, 48, and 72 hours.


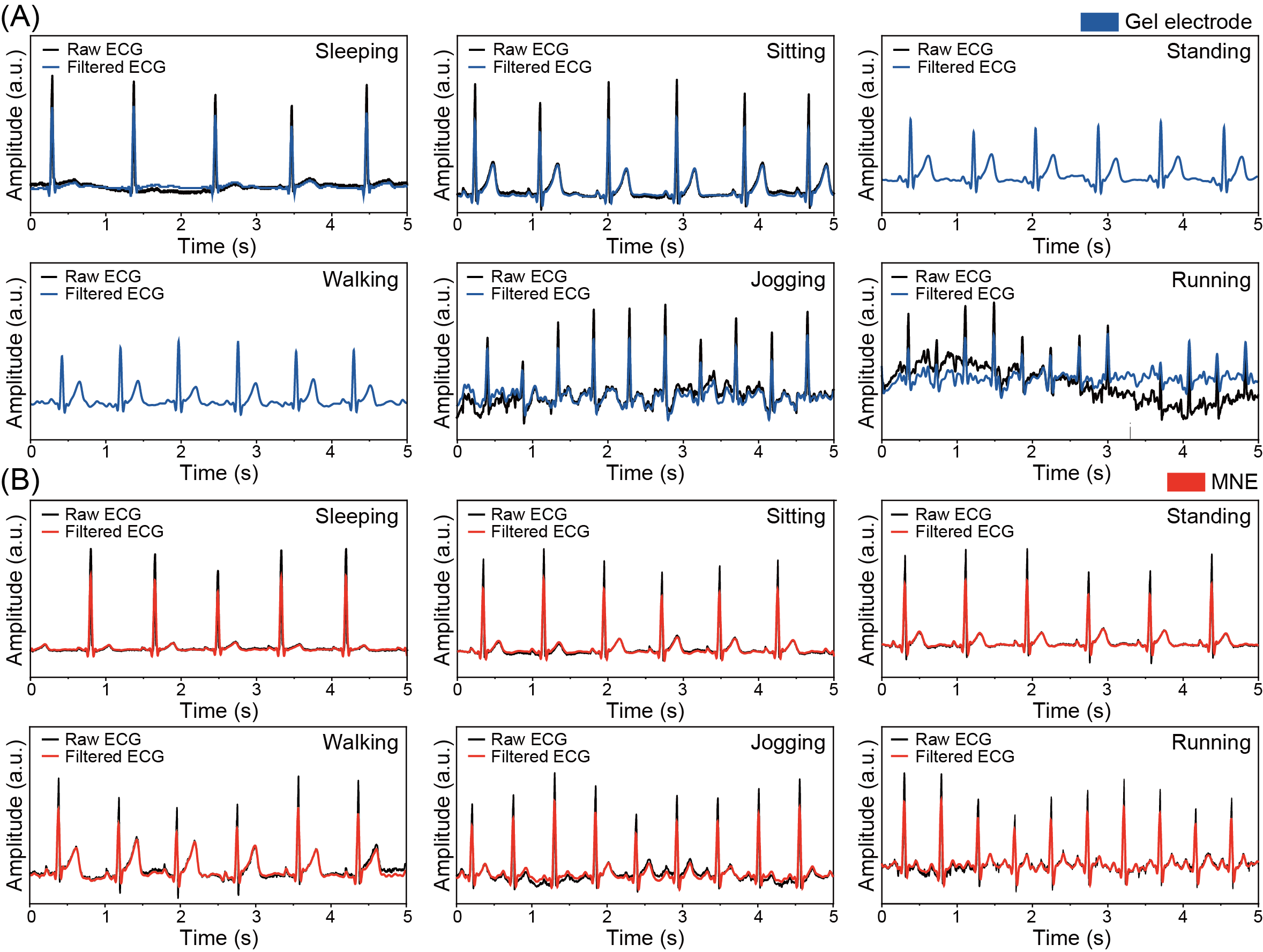


**Figure S9.** ECG signals were recorded using both gel and MNE during different motion states on day 1. The data were processed using a high-pass filter (0.5 Hz), a low-pass filter (20 Hz), and a band-stop filter (59–61 Hz).

**
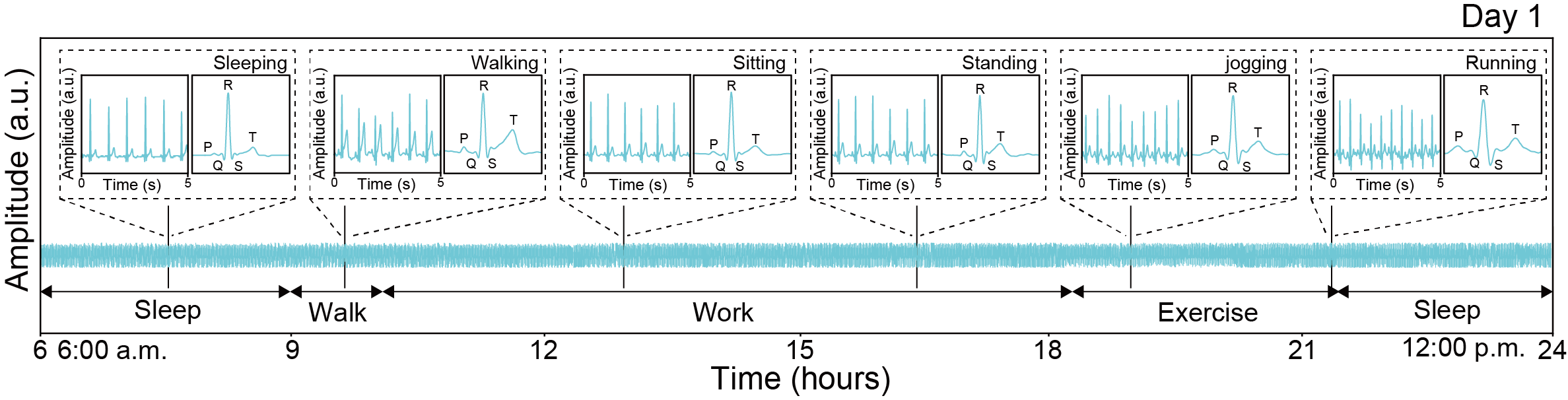
Figure S10.** Real-time 24-hour ECG data on the first day. The data was obtained after that microneedle was established on the chest of researcher immediately.

**
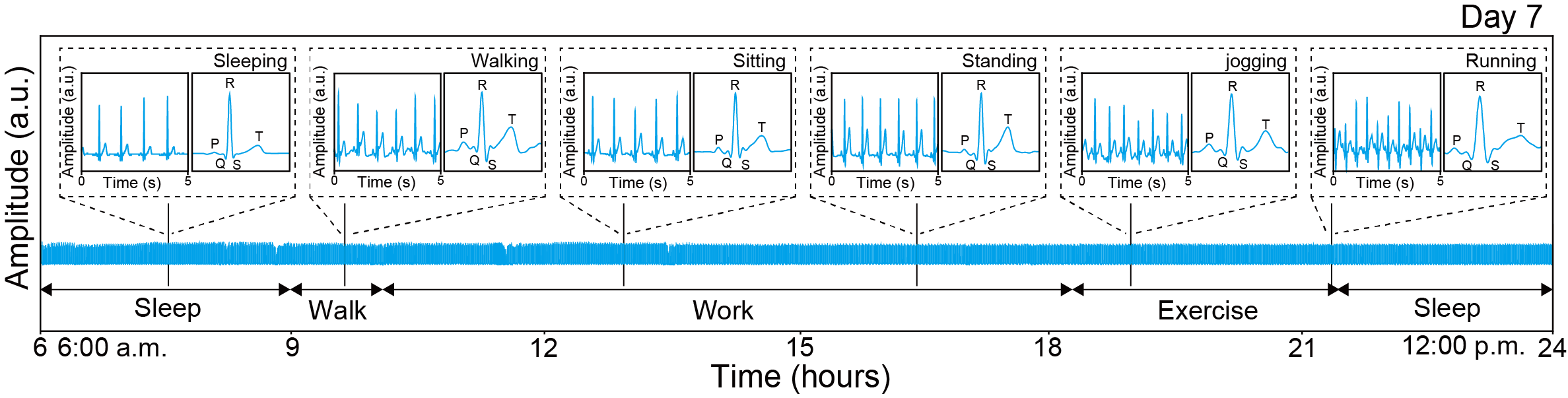
**

**Figure S11.** Real-time 24-hour ECG data after 7 days. Microneedle was established on the chest of researcher continuously.


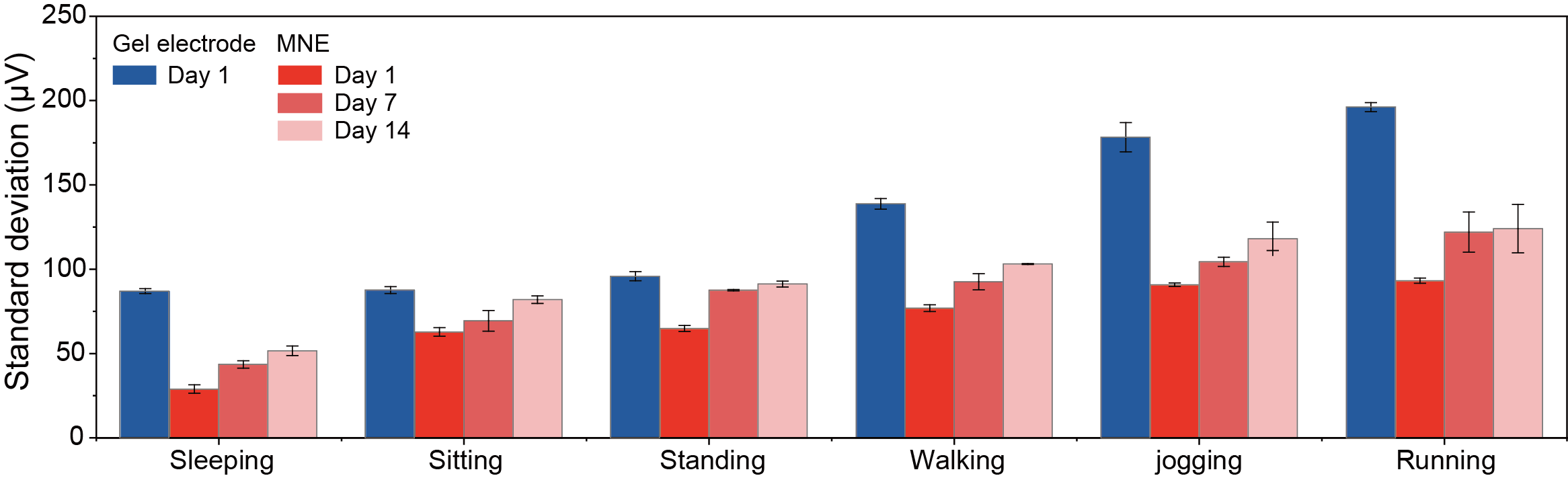


**Figure S12.** Standard deviation of baseline noise for gel electrodes and MNEs. Gel electrodes exhibited higher noise levels compared to MNEs, even after long-term use (14 days).


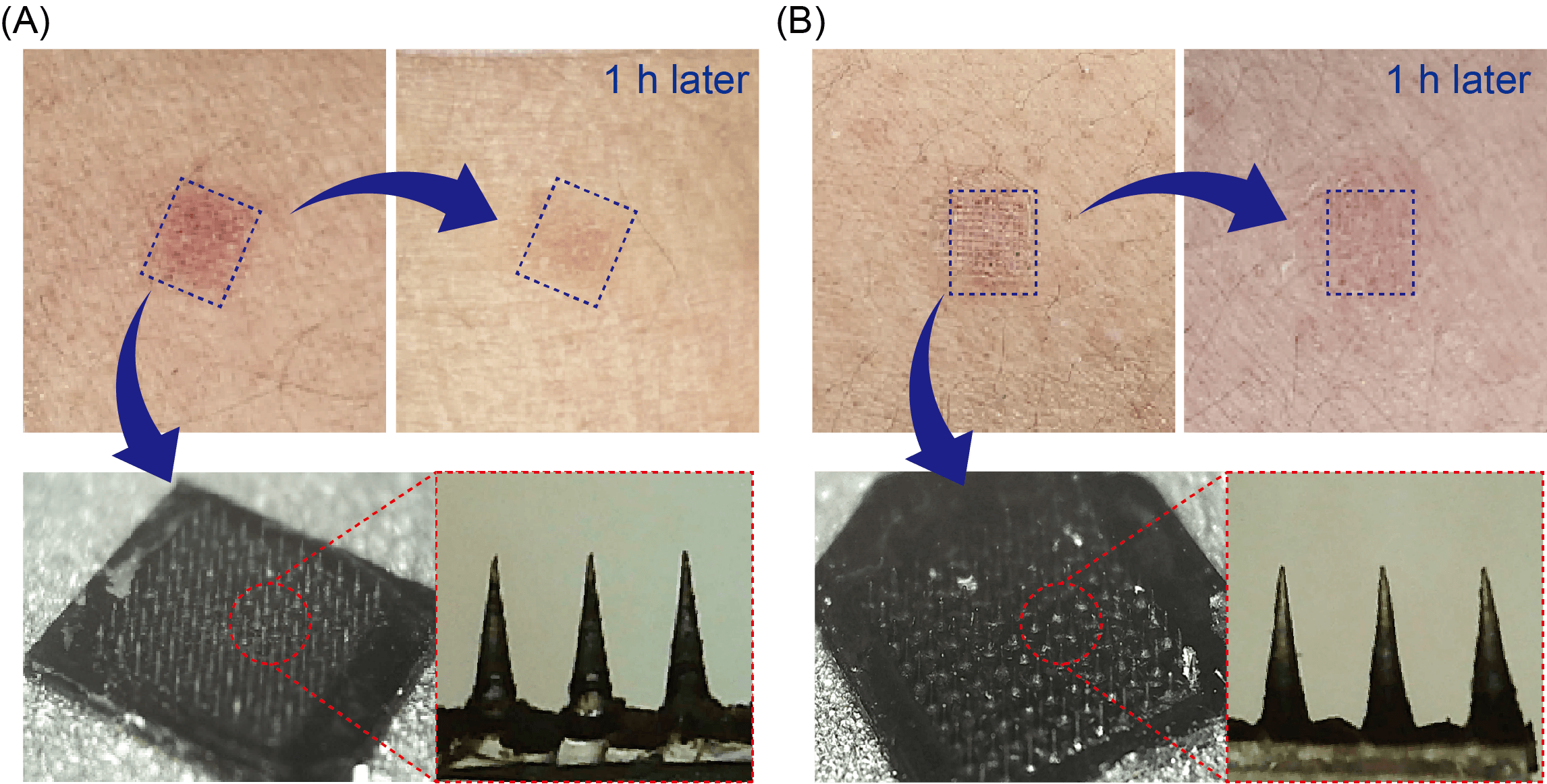


**Figure S13.** Skin condition and MNE integrity following prolonged wear on the skin for (A) 7 days and (B) 14 days.


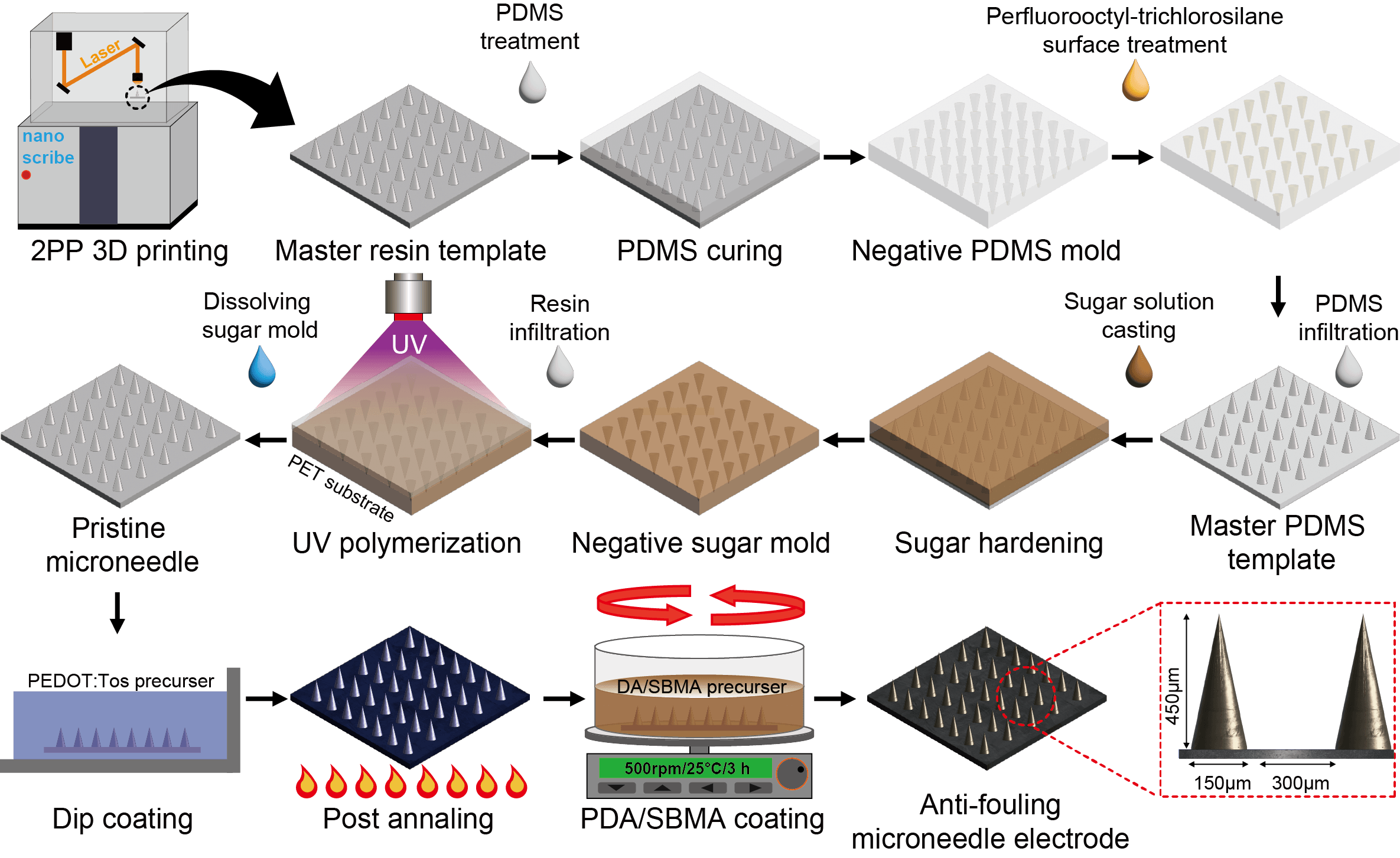


**Figure S14**. Schematic representing the fabrication process of MNs.

**
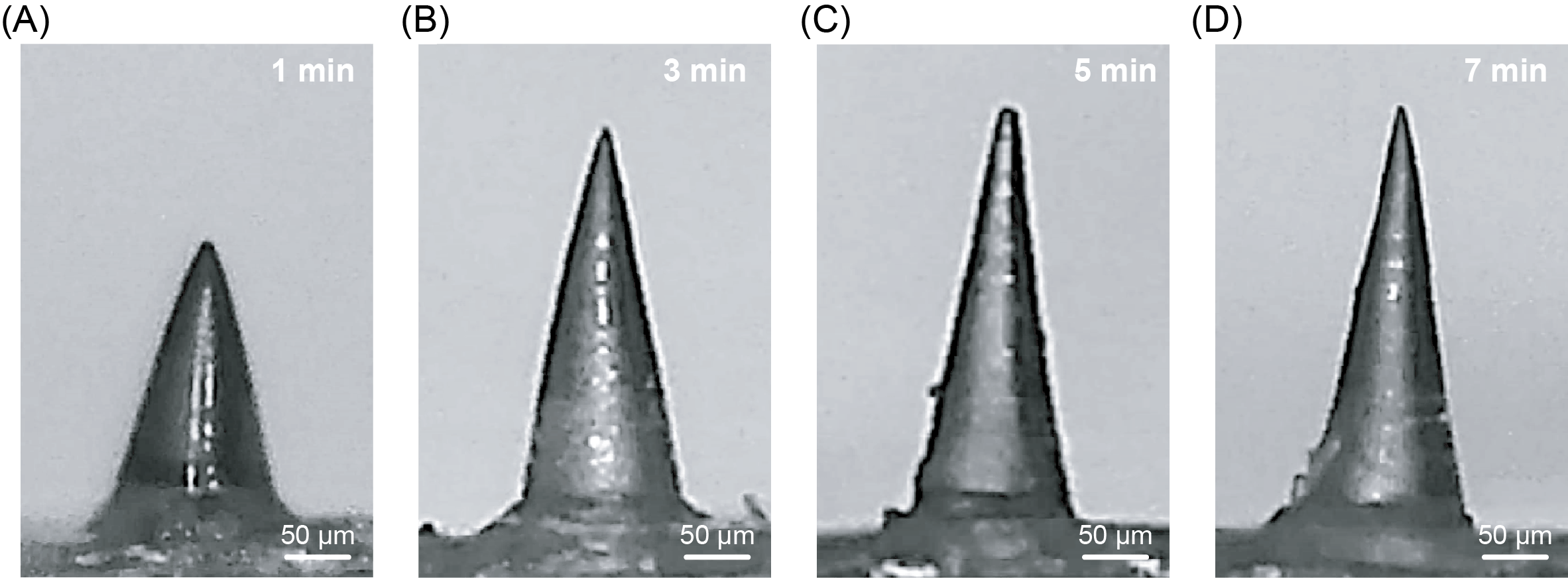
**

**Figure S15.** Optical images of microneedles fabricated under different UV curing times: (A) 1 min, (B) 3 min, (C) 5 min, and (D) 7 min.


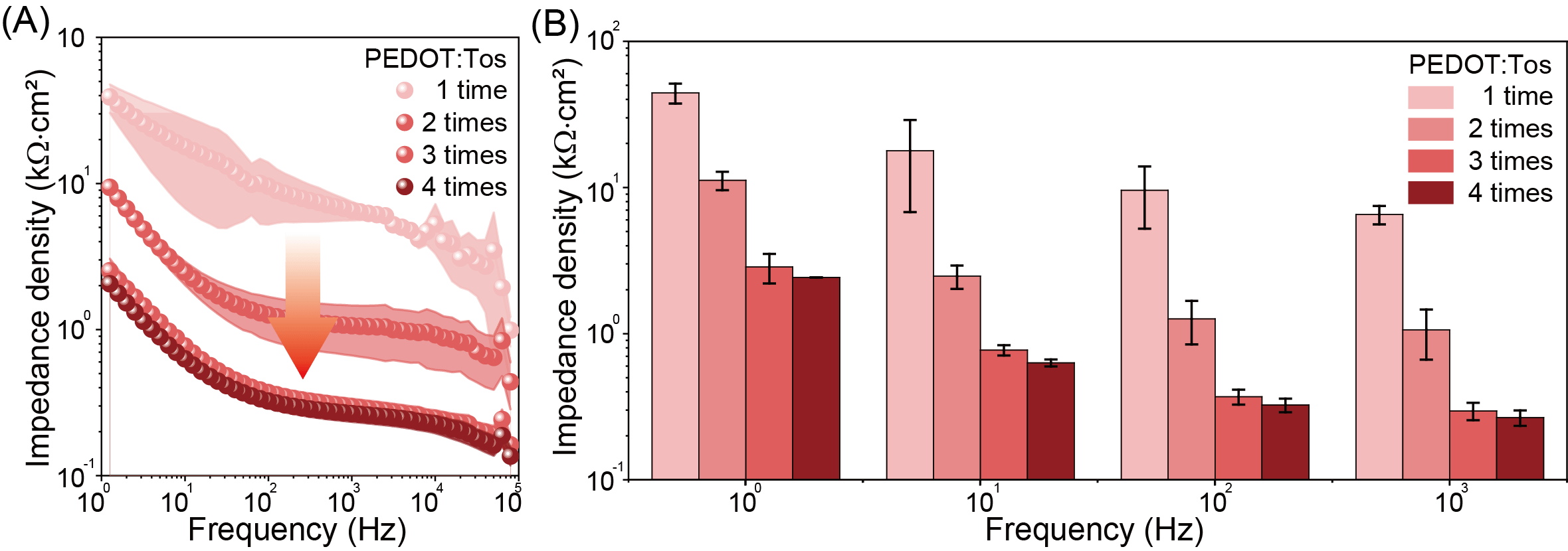


**Figure S16.** Impedance density of MNEs as a function of coating cycle. (A) impedance density spectra in accordance with PEDOT:Tos coating cycles from 1 to 4 times and (B) the corresponding impedances density at 1, 10, 100, and 1000 Hz.

**
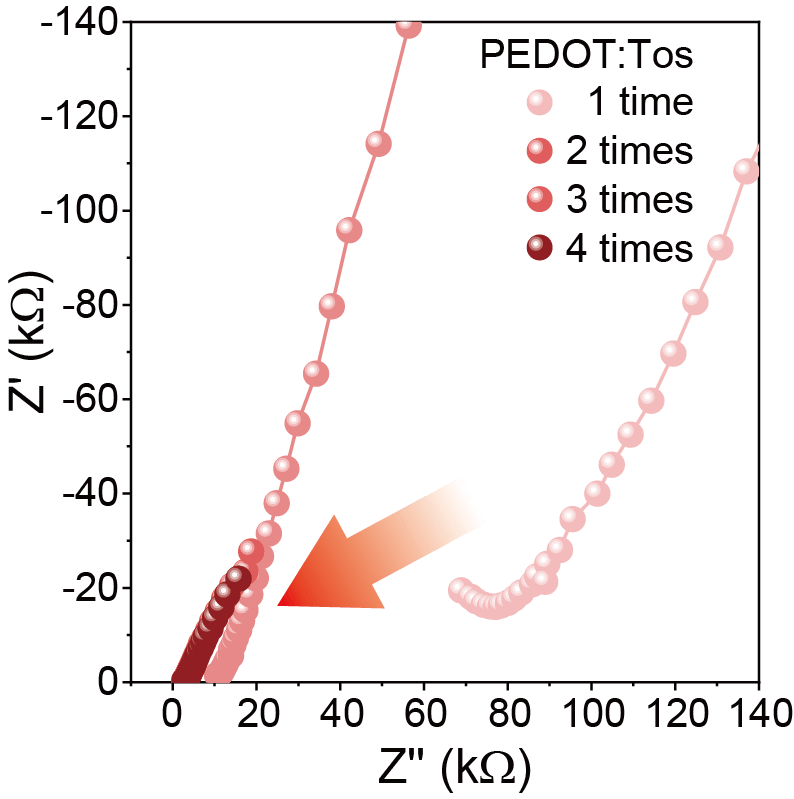
**

**Figure S17.** Nyquist plot of PEDOT:Tos-coated MNEs as a function of coating cycle. As the number of PEDOT:Tos coating cycles increases, electrical conductance improves and charge transfer resistance decreases.

**
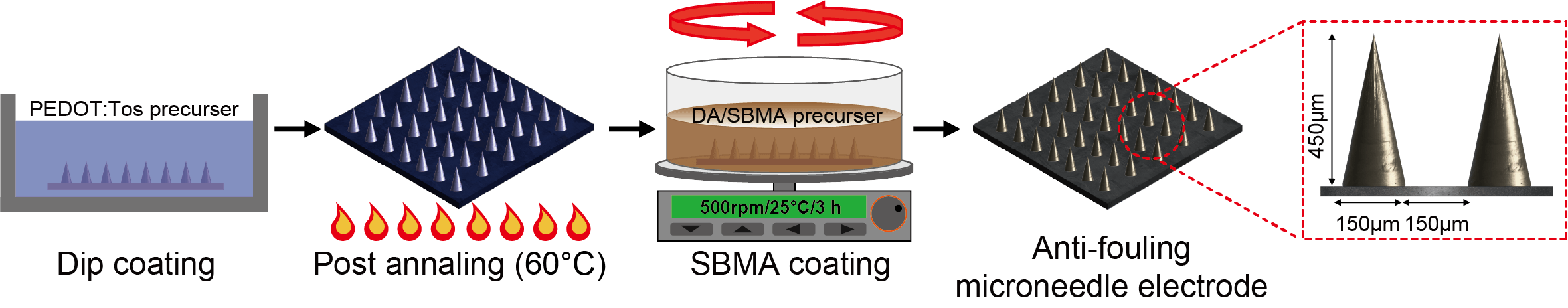
**

**Figure S18**. Coating process of MNEs. The PEDOT:Tos layer was deposited through a dip-coating process, while the SMBA layer was formed via a Michael addition reaction. Both methods are classified as wet processing techniques.


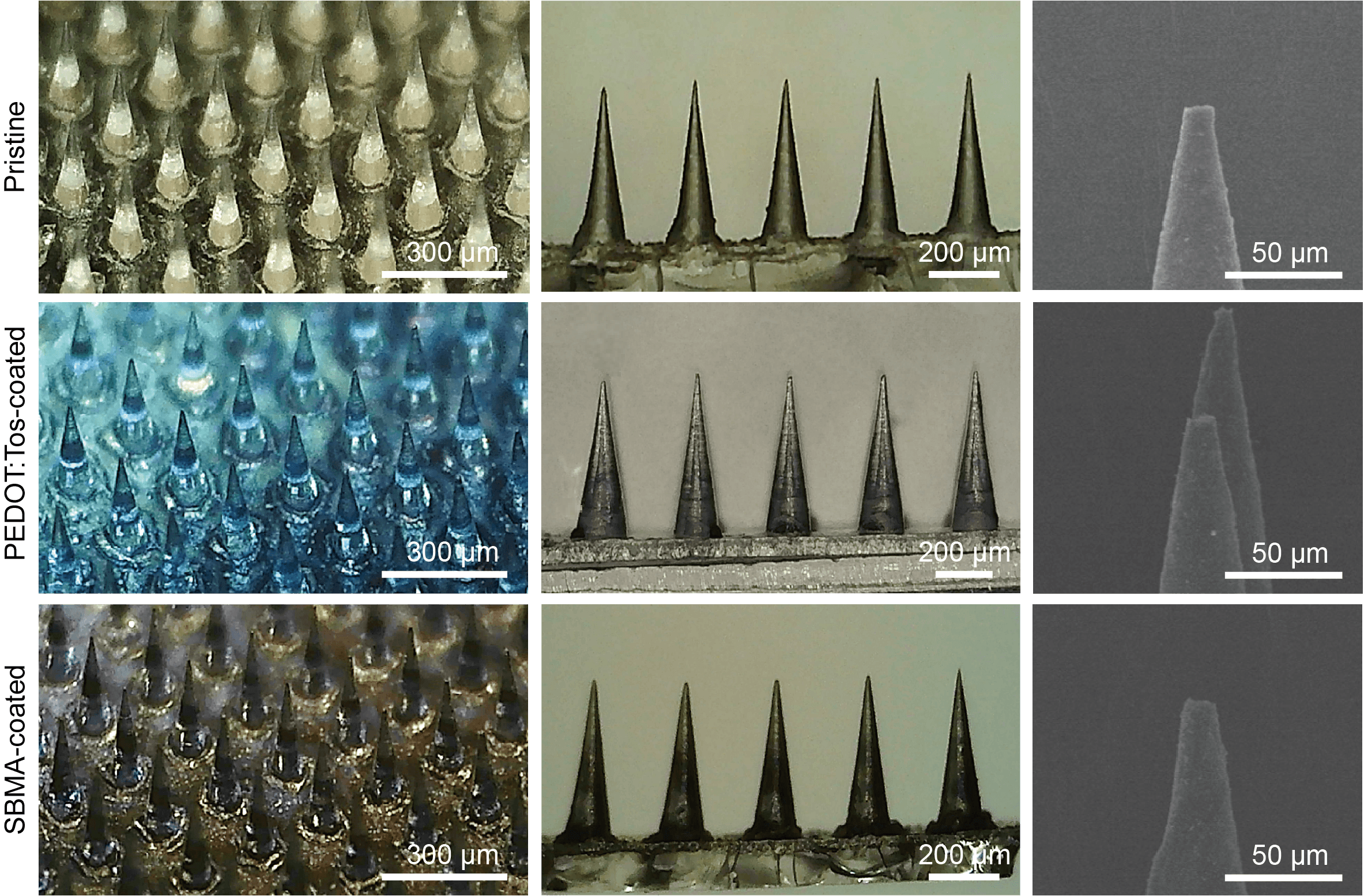


**Figure S19.** Optical image of MN (left) bird-eye’s view, (center) side view, and (right) SEM images of the MNE with different surface modification: (top) pristine, (center) PEDOT:Tos-coated and (bottom) SBMA-coated.


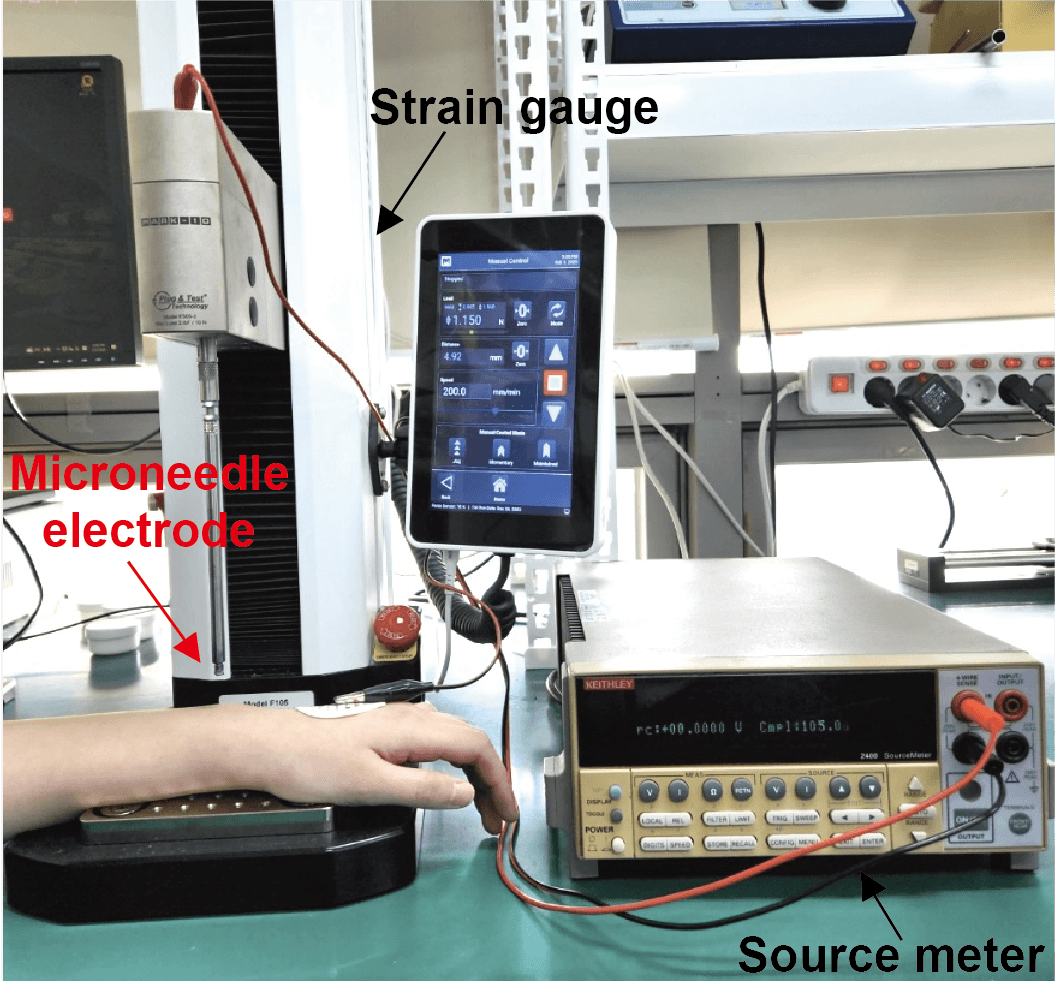


**Figure S20.** The setup for the insertion test on human skin involved a strain gauge and MNEs electrically connected in a closed loop with a gel electrode via a source meter.

**Figure S21.
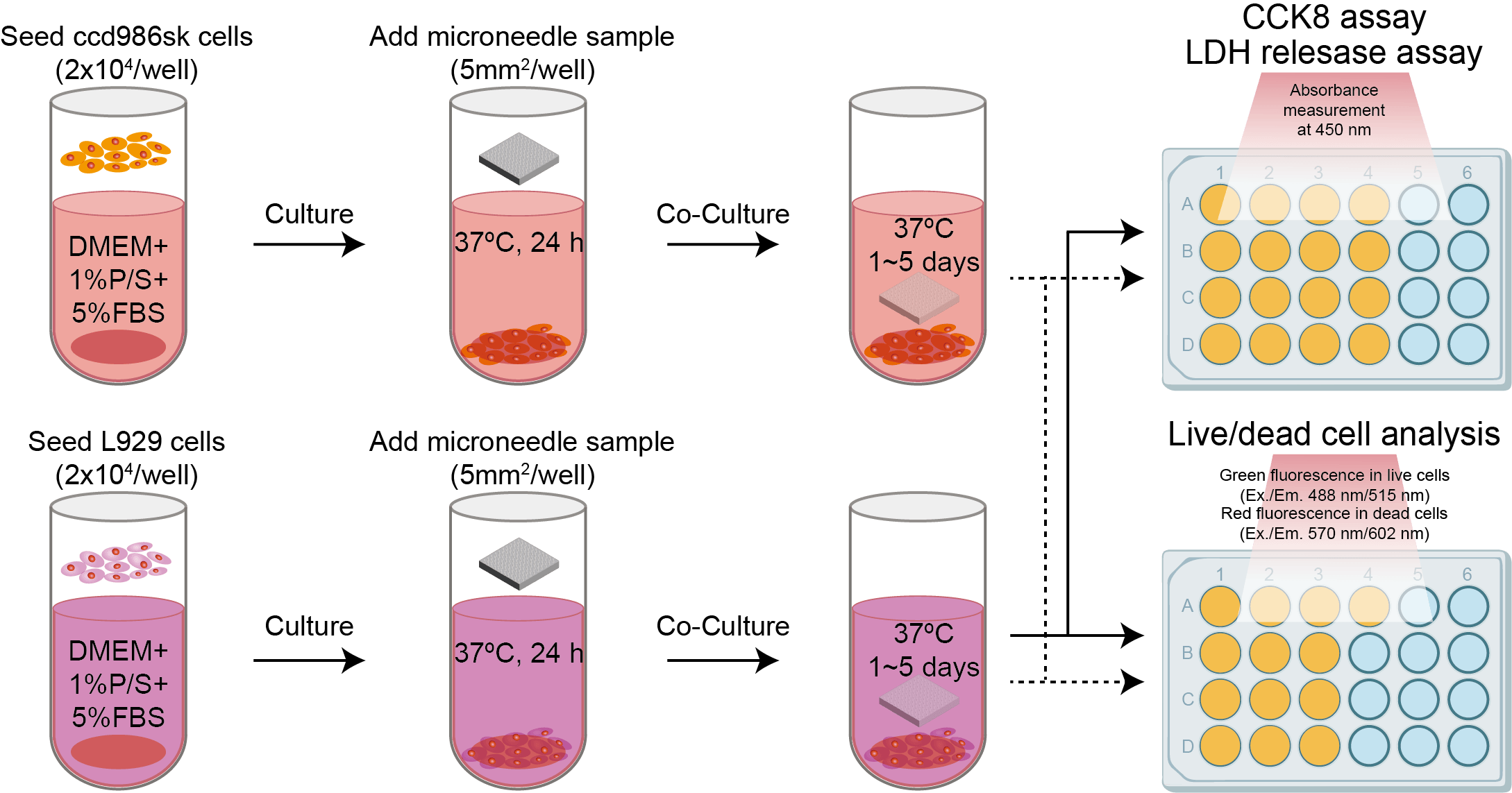
**Schematic diagrams illustrating the biocompatibility assessment of MNEs using L929 (mouse fibroblast cell line) and CCD-986sk (human skin fibroblast cell line).


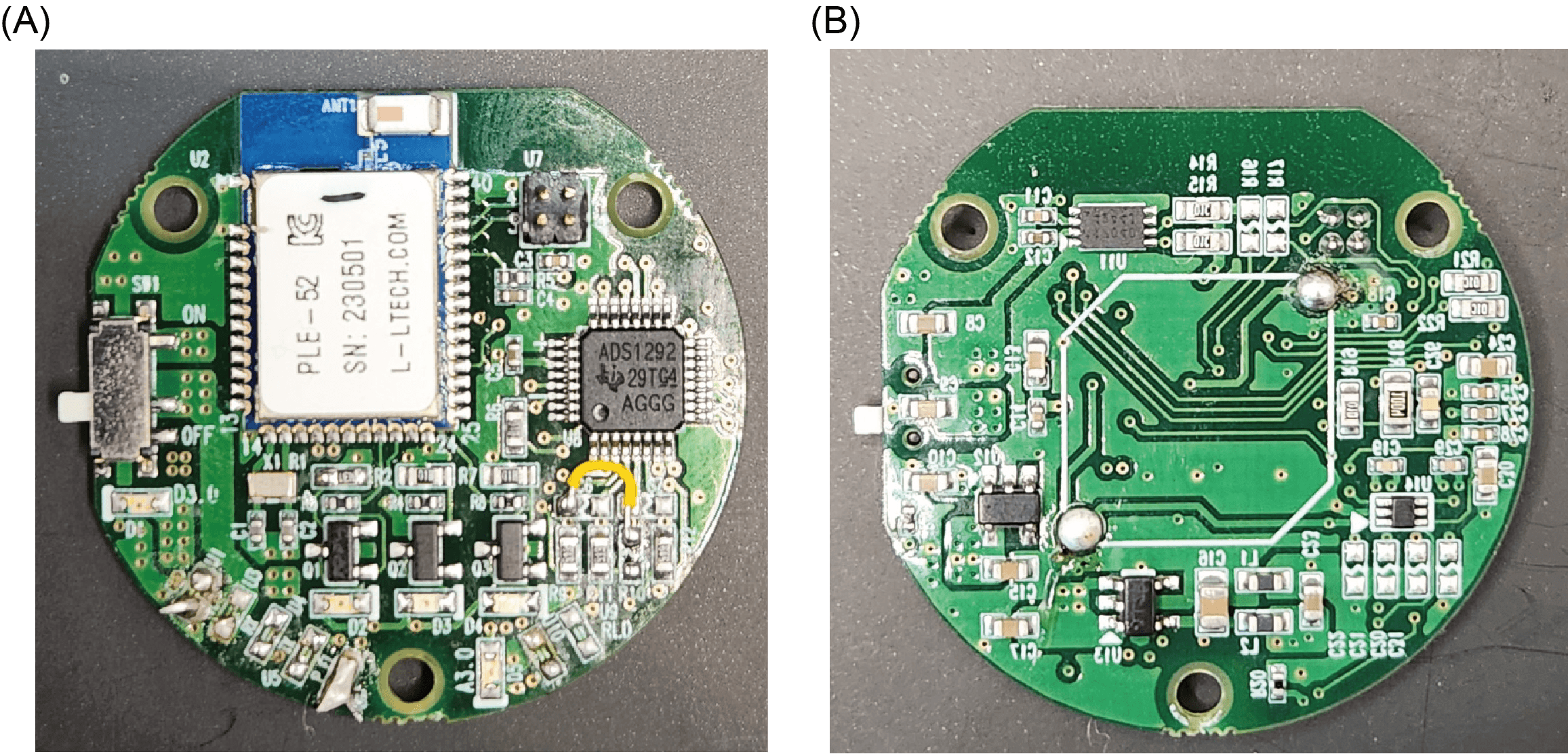


**Figure S22.** Optical images of the ECG monitoring circuit: (A) front view and (B) rear view of the circuit.


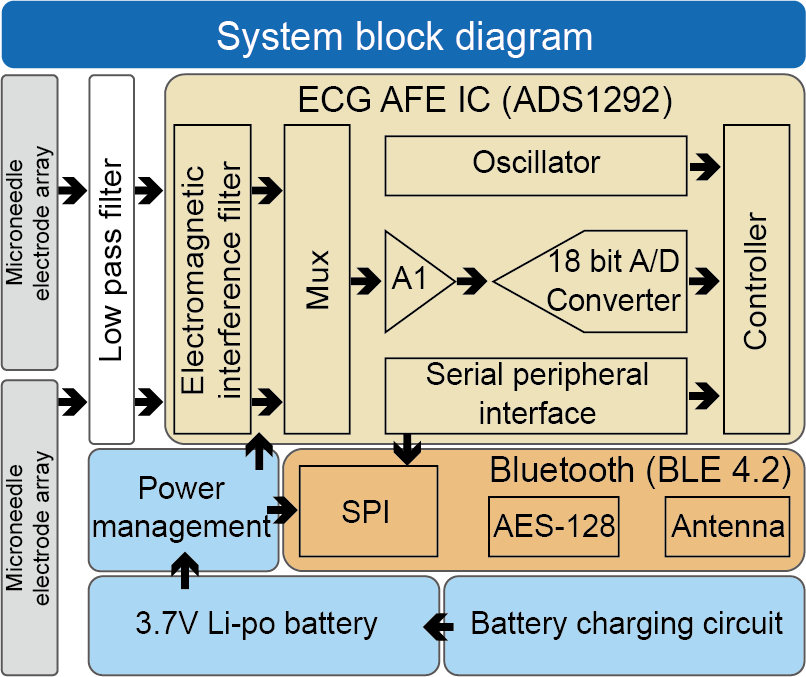


**Figure S23.** System block diagram of wireless ECG monitoring device.


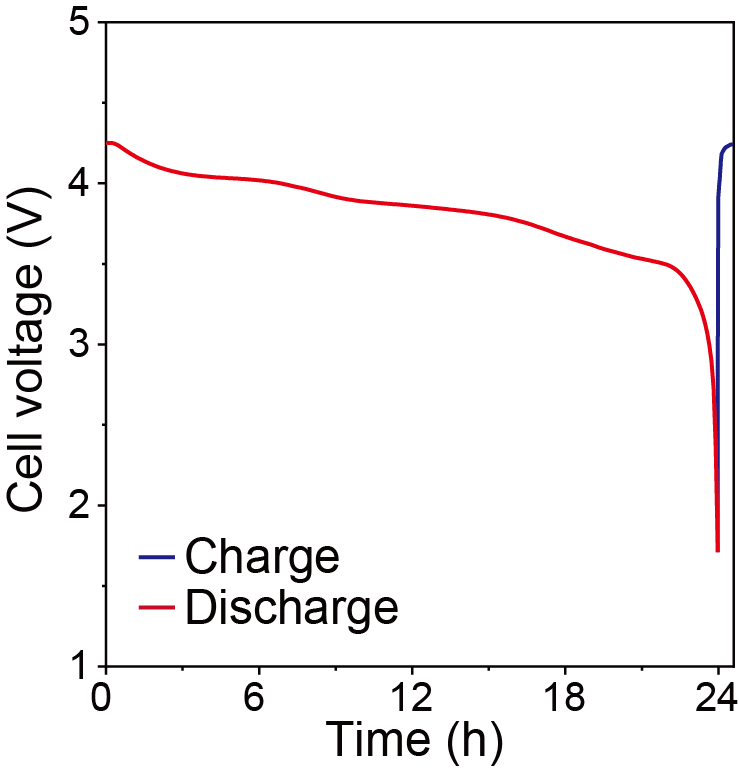


**Figure S24.** Battery discharge–charge test graph. The 220 mAh battery powers the system for up to 24 hours and takes approximately 40 minutes to fully recharge.


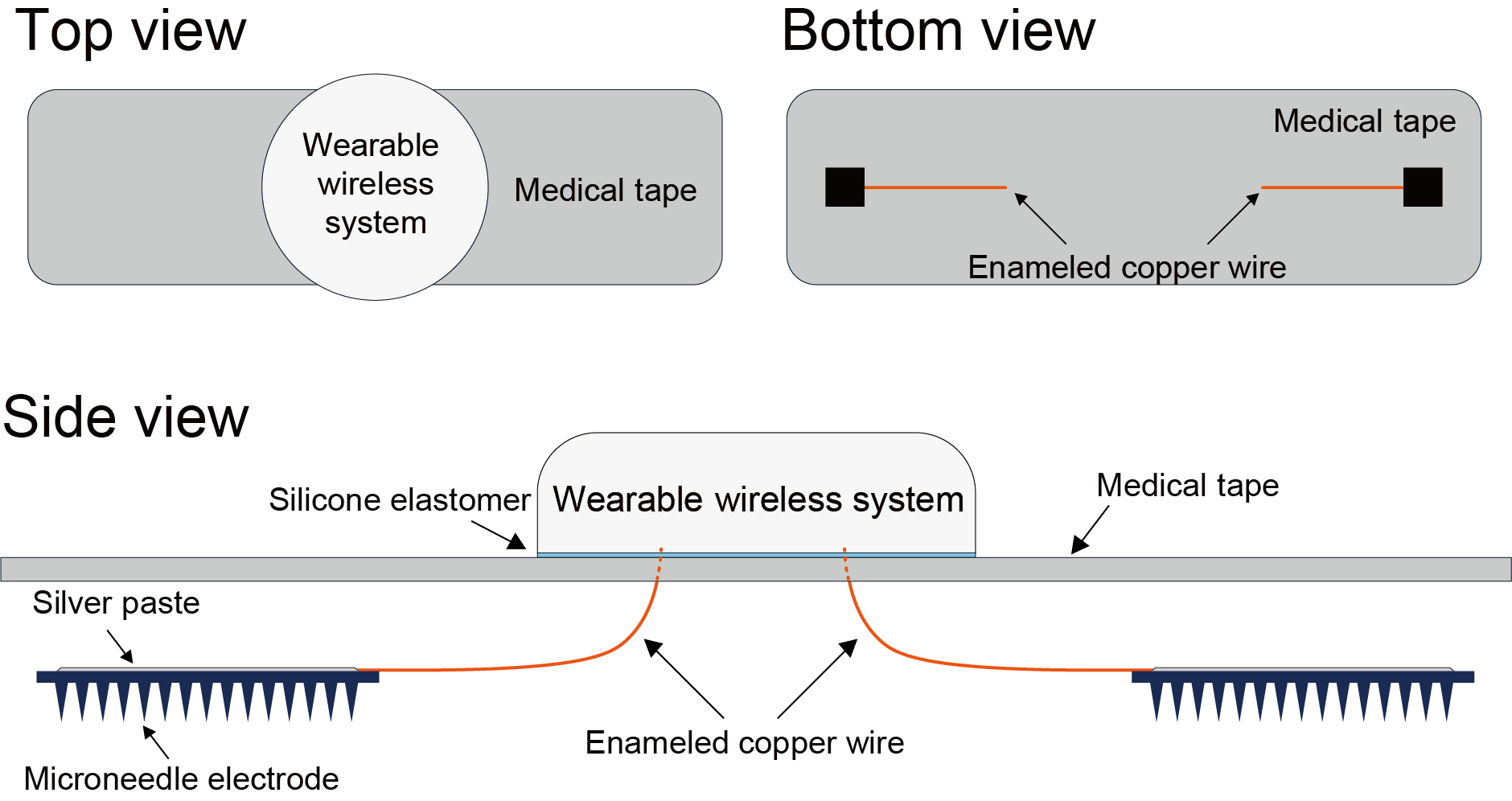


**Figure S25.** Schematic illustration of the wearable wireless ECG monitoring system with MNEs.


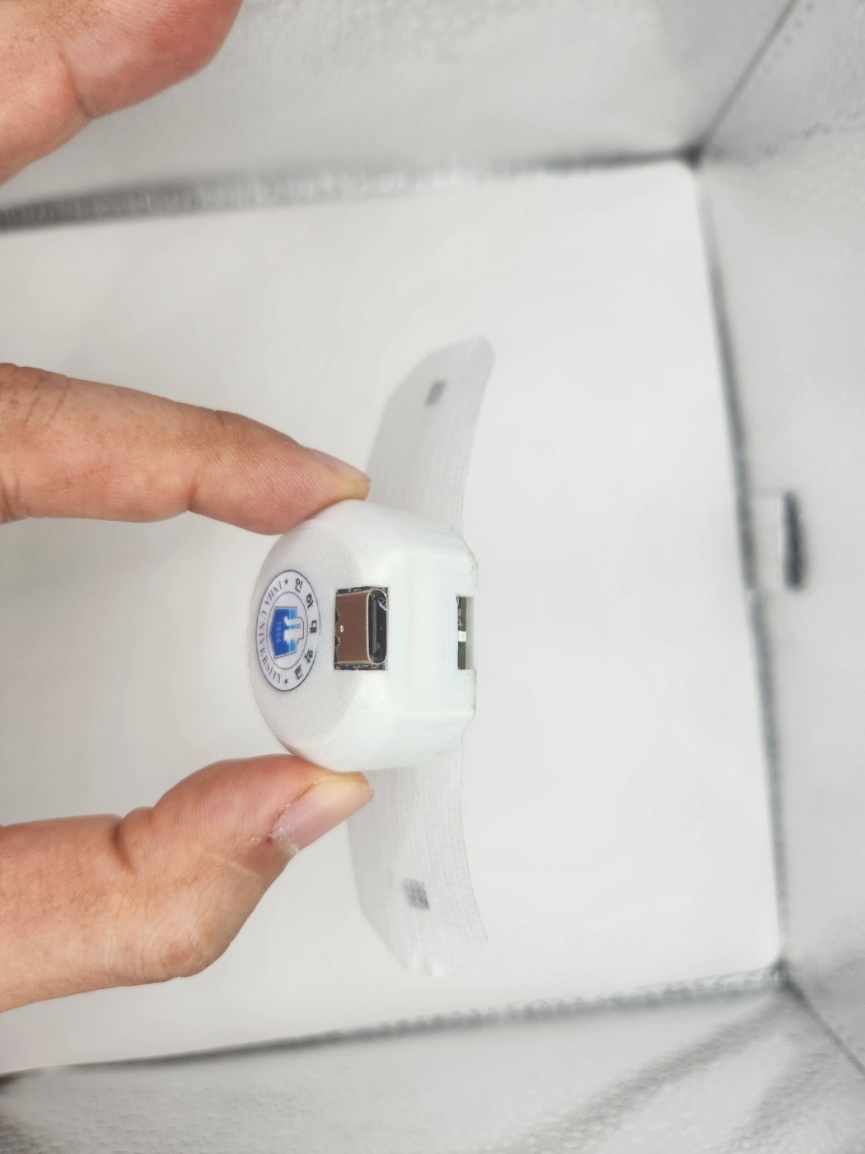


**Figure S****26**. Optical image of assembled ECG monitoring system.

**Table S1.** Penetration depths of representative SBMA-coated MNEs measured from the cross-sectional OCT image shown in Figure S6.

| No. | 1 | 2 | 3 | 4 | 5 | 6 | 7 | 8 | 9 | Avg. |
| --- | --- | --- | --- | --- | --- | --- | --- | --- | --- | --- |
| Penetration depth (μm) | 355.05 | 348.77 | 382.02 | 373.16 | 323.28 | 356.53 | 395.32 | 373.88 | 342.49 | 361.17 |

**Table S2.** Comparison of the parameters of the ECG monitoring using MNEs.

| No. | Materials | Coating  material | Impedance density  (kΩ∙cm^2^) @ 10Hz | Length (μm) | Antifouling | Long-term monitoring | Ref. |
| --- | --- | --- | --- | --- | --- | --- | --- |
| 1 | TEGDMA/DUDMA | PEDOT:Tos | 0.63 | 450 | O | 14 days | This work |
| 2 | Epoxy | PEDOT:PSS | 42.9 | 800 | X | - | ^[1]^ |
| 3 | Epoxy/iron particle | Ti/Au | 50* | 600 | X | - | ^[2]^ |
| 4 | PEGDA/MAA | Au | 56.5 @ 20 Hz | 500 | X | - | ^[3]^ |
| 5 | Polyimide | Au nanowire | 80 | 600 | X | - | ^[4]^ |
| 6 | Epoxy | Ti/Au | 92.6 @ 20Hz | 500 | X |  | ^[5]^ |
| 7 | Acrylic resin | Ti/Au | 225.5* | 3000 | X | - | ^[6]^ |
| 8 | Epoxy | Ti/Au | 643.1* | 630 | X | - | ^[7]^ |

*Estimated

**References**

[1] C. Zhou, G. Yao, X. Gan, K. Chai, P. Li, J. Peng, T. Pan, M. Gao, Z. Huang, B. Jiang, Z. Yan, K. Zhao, D. Yao, K. Chen, Y. Lin, *Npj Flex. Electron.* **2025**, *9*, 77.

[2] L. Ren, Q. Jiang, Z. Chen, K. Chen, S. Xu, J. Gao, L. Jiang, *Sens. Actuators Phys.* **2017**, *268*, 38.

[3] C.-W. Dong, C.-J. Lee, W.-T. Park, *Adv. Mater. Technol.*, e00812.

[4] L. Xing, L. Liu, R. Jin, H. Zhang, Y. Shen, S. Zhang, Z. He, D. Li, H. Ren, Q. Huang, X. Cao, S. Zhang, S. Dong, W. Cheng, B. Zhu, *ACS Appl. Mater. Interfaces* **2024**, *16*, 57695.

[5] O. P. Singh, A. Bocchino, T. Guillerm, Y. Hu, F. Stam, C. O’Mahony, *Adv. Mater. Technol.* **2024**, *9*, 2301606.

[6] P. Salvo, R. Raedt, E. Carrette, D. Schaubroeck, J. Vanfleteren, L. Cardon, *Sens. Actuators Phys.* **2012**, *174*, 96.

[7] Y. Hou, Z. Wang, H. Yu, *Microsyst. Nanoeng.* **2021**, *7*, 53.
